# Integrated Single-Cell Transcriptomics Identifies γδ T-Cell Heterogeneity and a Candidate HLA-E–NKG2A Regulatory Axis in Pancreatic Ductal Adenocarcinoma

**DOI:** 10.3390/cancers18111723

**Published:** 2026-05-25

**Authors:** Saikat Mandal, Shirin R. Hasan, Arkadeep Dhali, Manideepa Maji

**Affiliations:** 1 Nottingham Digestive Diseases Centre, Translational Medical Sciences, School of Medicine, University of Nottingham, Nottingham NG7 2UH, UK; 2 NIHR Nottingham Biomedical Research Centre, Nottingham University Hospitals NHS Trust and the University of Nottingham, Nottingham NG7 2UH, UK; 3 Queen’s Centre for Oncology and Haematology, Hull University Teaching Hospitals NHS Trust, Hull HU3 2JZ, UK; 4 Academic Unit of Gastroenterology, Sheffield Teaching Hospitals NHS Foundation Trust, Sheffield S5 7AU, UK; 5 School of Medicine, Dentistry and Biomedical Sciences, Queen’s University Belfast, Belfast BT9 7BL, UK; 6 Hull York Medical School, University of Hull, Hull HU6 7RX, UK; 7 Haematology, Hull University Teaching Hospitals NHS Trust, Cottingham, Hull HU16 5JQ, UK

**Keywords:** pancreatic ductal adenocarcinoma, gamma-delta T-cells, NKG2A, HLA-E, tumour microenvironment, chemokines single-cell RNA sequencing

## Abstract

**Background:** Pancreatic ductal adenocarcinoma (PDAC) has a profoundly immunosuppressive tumour microenvironment (TME) that limits anti-tumour immunity and contributes to resistance to immunotherapy. Although γδ T-cells can integrate innate and adaptive immune signals, their abundance, transcriptional states and regulatory pathways in PDAC remain incompletely defined. **Methods:** We performed integrated single-cell RNA sequencing analysis of 39 pancreatic tissue samples, comprising 33 PDAC tumours and 6 adjacent normal tissues. After dataset integration, immune cell annotation, and stringent per-cell gating, γδ T-cells were quantified and profiled for checkpoint-, ligand-, and chemokine-related programmes. **Results:** γδ T-cells were detectable across PDAC samples but showed substantial inter-sample heterogeneity in abundance. Among candidate inhibitory pathways, *PDCD1*, *CD274*, and *HAVCR2* expression in γδ T-cells did not differ significantly between tumour and adjacent tissues, whereas *KLRC1* (encoding NKG2A) showed a tumour-associated difference at the single-cell level, with a consistent directional pattern in sample-level summaries. NKG2A expression was comparable between γδ T-cells and NK cells, suggesting a shared inhibitory programme. HLA-E, the ligand for NKG2A, showed higher epithelial-cell expression in tumour than adjacent tissue in sample-level summaries (median 1.06 vs. 0.57; BH-q = 0.035). Chemokine analysis identified enrichment of CCL2, CCL4, CCL5, CXCL8 and CXCL12, with limited CXCL9/10/11 signalling within the PDAC TME. Within γδ T-cells, CXCR4 was the trafficking receptor, followed by CCR6, CCR7 and CXCR6. **Conclusions:** PDAC-infiltrating γδ T-cells show marked inter-sample heterogeneity and variable inhibitory and trafficking-related programmes. Integrated transcriptomic analysis nominates HLA-E–NKG2A as a candidate regulatory axis, with NK cells included as a biologically relevant comparator. Chemokine receptor patterns, particularly CXCR4, CCR6, CCR7 and CXCR6, suggest candidate trafficking features. These findings are hypothesis-generating and require spatial, protein-level and functional validation.

## Simple Summary

Pancreatic cancer is difficult to treat partly because the tumour environment can weaken immune responses. In this study, we analysed publicly available single-cell data from pancreatic cancer samples to better understand a group of immune cells called γδ T-cells. These cells were present in pancreatic tumours, but their numbers varied widely between patients. We also found signals suggesting that these cells, together with natural killer cells, may be influenced by an immune-control pathway involving HLA-E and NKG2A. In addition, we identified chemokine patterns that may help explain how these immune cells are recruited or retained within pancreatic tumours. These findings provide a starting point for future studies using laboratory, spatial and protein-level methods to confirm how these immune pathways operate in pancreatic cancer.

## 1. Introduction

Pancreatic ductal adenocarcinoma (PDAC) is the most common form of pancreatic cancer and remains one of the deadliest solid malignancies worldwide [1,2]. The poor prognosis associated with PDAC is largely attributed to late-stage diagnosis due to non-specific early symptoms and the absence of reliable screening markers, limiting the proportion of patients eligible for curative surgical resection [3]. Current treatment strategies include surgical resection when feasible and combination chemotherapy regimens such as FOLFIRINOX or gemcitabine plus nab-paclitaxel. However, therapeutic responses remain limited [4]. A major contributor to treatment resistance is the highly immunosuppressive and desmoplastic tumour microenvironment (TME), which restricts effective anti-tumour immune responses and promotes disease progression [5,6].

The PDAC immune microenvironment is enriched in immunosuppressive cell populations that promote tumour progression. Myeloid-derived suppressor cells (MDSCs) and M2-polarised tumour-associated macrophages contribute to adaptive immune suppression and tumour progression [7,8,9]. In contrast, infiltration of cytotoxic CD8^+^ T-cells and CD4^+^ Th1 cells has been associated with improved patient survival, whereas intratumoral CD4^+^ Th2 cells and regulatory T-cells (Tregs) correlate with poorer outcomes [10,11].

Among immune populations within the TME, γδ T-cells have emerged as important regulators of tumour immunity due to their ability to integrate innate and adaptive immune signalling [12,13]. However, their abundance and functional roles appear highly heterogeneous across PDAC cohorts, suggesting that tumour-intrinsic and microenvironmental cues may influence their recruitment and activity [13,14,15]. In addition to potential cytotoxic functions, γδ T-cells in PDAC have also been reported to suppress conventional alpha–beta (αβ) T-cell responses through expression of checkpoint ligands such as PD-L1 and Galectin-9, highlighting their context-dependent roles within the TME [14,16].

γδ T-cells express a diverse repertoire of receptors that enables them to recognise cellular stress and transformed cells independently of classical major histocompatibility complex (MHC) presentation [12,17]. Their defining γδ T-cell receptor (TCR) recognises non-peptide antigens and stress-induced ligands, while additional receptors, including activating natural killer receptors such as NKG2D and inhibitory receptors such as NKG2A, further modulate their cytotoxic responses [18,19,20]. Through these receptors, γδ T-cells can rapidly respond to cellular stress signals and contribute to tumour immune surveillance [12,21]. However, their activity is tightly regulated by immune checkpoint pathways.

One such pathway involves the interaction between the inhibitory receptor NKG2A and the non-classical MHC class I molecule human leucocyte antigen-alpha chain E (HLA-E) [20,22]. Engagement of HLA-E with NKG2A/CD94 on cytotoxic lymphocytes, including NK cells and γδ T-cells, delivers inhibitory signals that suppress cytotoxicity and cytokine production [23,24]. This pathway is best characterised in NK-cell biology, where HLA-E recognition by the NKG2A/CD94 receptor complex restrains cytotoxic responses; therefore, NK cells provide a biologically relevant comparator when assessing whether γδ T-cells may share this inhibitory programme in PDAC. Tumours can exploit this mechanism, particularly when classical HLA class I expression is reduced, to evade T-cell- and NK-cell-mediated immune responses [23].

In parallel with checkpoint signalling, immune cell localisation within tumours is regulated by chemokine networks [25,26,27]. γδ T-cells express several chemokine receptors, including CCR5, CCR6, CXCR3 and CXCR4, which guide their migration toward inflammatory chemokines [12,18]. The PDAC TME contains a complex chemokine landscape produced by tumour cells, cancer-associated fibroblasts, pancreatic stellate cells and infiltrating immune cells [28]. Key chemokines such as CCL2, CCL5, CXCL8, CXCL9, CXCL10 and CXCL12 regulate the recruitment of monocytes, macrophages and lymphocytes [17,29]. In particular, the CXCL12–CXCR4 axis contributes to immune exclusion by limiting effector T-cell infiltration into tumour nests, while CCL2 and CCL5 promote recruitment of immunosuppressive myeloid populations [30,31]. These chemokine gradients may therefore influence the spatial organisation and functional state of tumour-infiltrating γδ T-cells within the PDAC microenvironment.

Recent advances in single-cell transcriptomic technologies have enabled high-resolution profiling of immune populations within complex tumour ecosystems [32,33,34]. Such approaches provide an opportunity to resolve the heterogeneity of tumour-infiltrating lymphocytes and to identify regulatory pathways shaping their functional states. However, the transcriptional landscape and regulatory programmes of γδ T-cells within PDAC remain incompletely characterised. Previous studies have shown that γδ T-cells can have context-dependent roles in PDAC, ranging from cytotoxic anti-tumour activity to suppression of αβ T-cell responses. Separately, the HLA-E–NKG2A pathway has emerged as an inhibitory checkpoint in cytotoxic lymphocytes, including NK cells and γδ T-cells, but its relationship to γδ T-cell states in PDAC remains incompletely defined. The novelty of the present study is the integrated analysis of multiple public PDAC single-cell RNA-sequencing (scRNA-seq) cohorts to jointly assess γδ T-cell abundance, *KLRC1*/NKG2A expression, epithelial HLA-E expression and chemokine programmes within the same analytical framework.

Here, we performed an integrated analysis of publicly available scRNA-seq datasets to define the transcriptional features of tumour-infiltrating γδ T-cells in PDAC. By comparing tumour and adjacent tissues across multiple cohorts, we aimed to characterise the abundance and heterogeneity of γδ T-cells and identify candidate regulatory pathways that influence their function. In particular, we focused on inhibitory checkpoint signalling and chemokine-mediated trafficking programmes that may shape γδ T-cell activity within the pancreatic TME.

## 2. Methods

### 2.1. Data Sources and Cohort Selection

Publicly available scRNA-seq datasets for PDAC were analysed to identify tumour-infiltrating γδ T-cells and characterise immune regulatory molecules within the TME. Four Gene Expression Omnibus (GEO) cohorts were processed (GSE212966 [32], GSE217845 [33], GSE279781 [34], and GSE214295 [35]), comprising 39 samples in total: 33 PDAC tumours and 6 adjacent normal pancreatic tissue samples. Patient-derived organoid (PDO) samples were excluded. Where adjacent/normal tissue was provided, it was retained for tumour versus adjacent contrasts.

### 2.2. Data Loading and Quality Control

All analyses were performed in R version 4.5.1 (R Foundation for Statistical Computing, Vienna, Austria) using Seurat (v5.0) [36,37]. For each sample, count matrices were imported from 10X-format files (matrix.mtx, barcodes.tsv, features.tsv/genes.tsv) or count tables. Gene identifiers were harmonised across cohorts (gene symbol selection where present and unique feature naming), and barcode-matrix concordance was verified. Quality control of the scRNA-seq data retained cells with at least 200 detected genes (nFeature_RNA) and 500 unique molecular identifiers (nCount_RNA) to remove low-complexity transcriptomes. Cells with an unusually low gene-to-UMI ratio were excluded as an additional conservative quality-control step to reduce low-complexity or potentially artefactual transcriptomes. This threshold was not used as a formal doublet-calling method; therefore, residual doublet contamination cannot be fully excluded and is acknowledged as a limitation. Additional filtering removed cells with more than 20% mitochondrial transcripts, indicative of poor-quality or stressed cells, and those with haemoglobin transcript levels above 3% to minimise red blood cell contamination.

### 2.3. Normalisation, Integration and Clustering

Quality-filtered samples were merged into a single Seurat object while retaining dataset, sample identifier, tissue label and patient identifier metadata. Expression was log-normalised (LogNormalize; scale factor 10,000), and 3000 highly variable genes were selected using the variance-stabilising transformation (vst). Data were scaled (ScaleData), and principal component analysis (PCA) was performed using 50 PCs. Batch effects were addressed using Harmony with the dataset as the integration variable (PCs 1-30). Nearest-neighbour graphs and UMAP [38,39] embeddings were constructed on the Harmony reduction (dims 1-30). Louvain clustering was performed (FindClusters) at resolution 0.9. Cluster markers were identified using FindAllMarkers (Wilcoxon rank-sum; min.pct = 0.25; logfc.threshold = 0.25; max 500 markers per cluster).

### 2.4. Cell Type Annotation and γδ T-Cell Identification

Cell types were assigned using a hierarchical marker-based strategy: major compartments were annotated as epithelial, endothelial, fibroblast or pericyte, and immune (PTPRC-positive), then refined into immune lineages using canonical markers. To precisely identify γδ T-cells and minimise misclassification with αβ T-cells and NK cells, a per-cell gating strategy was applied as a final override. Cells were labelled as γδ T-cells if at least five of six core genes (*PTPRC*, *CD3D*, *CD3E*, *TRDC*, *TRGC1*, *TRGC2*) were expressed above 0.5 (log-normalised), *CD8A* and *CD8B* were below 0.5, and CD4 and FGFBP2 were below 0.05. NK cells were identified separately by requiring expression of at least two of *FGFBP2*, *KLRF1*, *NKG7* and GNLY (>0) with low T-cell gene expression (*CD3D* and *CD3E* below 0.1) [40,41]. Sensitivity analysis confirmed that per-sample γδ T-cell abundance was robust to modest perturbations in gating thresholds (Spearman ρ ≥ 0.997 across the perturbation grid; Supplementary Table S1). The γδ T-cell *KLRC1*/NKG2A expression signal was also directionally preserved across the same threshold perturbation grid, indicating that the observed *KLRC1* finding was not driven by a single gating cut-off.

As an orthogonal annotation sensitivity analysis, γδ T-cells were also identified using the published TCR module-score approach of Song et al. [42]. Within the lymphoid compartment, γδ TCR signal was assessed using TRDC, and αβ TCR signal was assessed using TRAC, TRBC1 and TRBC2. Putative γδ T-cells were defined by γδ TCR-high and αβ TCR-low module scores. *KLRC1*/NKG2A expression and γδ T-cell abundance were then compared with the primary strict gate used by us.

### 2.5. Quantification of Regulatory Molecules

Per-sample γδ T-cell and NK-cell infiltration was calculated as the proportion of all cells meeting the respective gating criteria (reported for tumour tissue). In γδ T-cells, expression of *PDCD1* (PD-1), *CD274* (PD-L1), *KLRC1* (NKG2A), *TIGIT*, *LAG-3* and *HAVCR2* (TIM-3) was quantified; *KLRC1* was also summarised in NK cells (percentage positive and mean expression per sample) to compare checkpoint utilisation between lineages. To assess epithelial ligands relevant to γδ T-cell regulation, epithelial cells were subset and analysed for classical MHC class I genes (HLA-A, HLA-B and HLA-C) and non-classical HLA genes (HLA-E, HLA-F and HLA-G).

To distinguish malignant-like epithelial cells from non-malignant epithelial cells, copy-number inference was performed using inferCNV [43] on epithelial clusters, with immune and stromal cells from the same sample used as reference populations. This analysis was used as a sensitivity assessment for epithelial HLA-E expression and is reported in Supplementary Tables S5 and S6.

### 2.6. Chemokine Ligand and Receptor Profiling

Chemokine ligands (CCL2-5, CCL7-8, CCL20, CCL27-28, CXCL5-6, CXCL8-12 and CXCL16) and chemokine receptors (CCR1-7, CCR10 and CXCR1-6) were quantified across the integrated atlas and summarised by mean expression and percentage-positive cells across annotated cell types and individual samples, with visualisation using UMAP feature plots and dot plots.

To complement gene-level ligand and receptor profiling, ligand–receptor inference was performed using two independent frameworks, CellChat [44] and LIANA [45], in samples with ≥10 γδ T-cells, comprising 19 tumour and 4 adjacent samples. CellChat was applied using the curated human ligand–receptor database, and LIANA was used to aggregate predictions across NATMI, Connectome, SingleCellSignalR, logFC and CellPhoneDB. Analyses focused on HLA-E–*KLRC1/KLRD1* interactions between epithelial, immune and stromal sender populations and γδ T-cell or NK-cell receiver populations; full settings are provided in the Supplementary Methods.

### 2.7. Statistics, Ethics and Reproducibility

Tumour versus adjacent comparisons were performed as exploratory analyses. For cell-level visualisations, Wilcoxon rank-sum tests were applied to log-normalised single-cell expression values and are labelled as cell-level analyses. To reduce the risk of inflated inference from treating cells as independent observations, key findings for *KLRC1*/NKG2A and HLA-E were additionally summarised at the sample level using per-sample mean expression and percentage-positive cells, followed by two-sided Wilcoxon rank-sum tests comparing tumour and adjacent normal samples. Tumour and adjacent samples were treated as unpaired because fully matched tumour–adjacent pairs were not available across all cohorts. *p*-values are reported as exploratory tests of pre-specified candidate molecules. Benjamini–Hochberg adjusted q-values were calculated within pre-defined gene families, including checkpoint genes, HLA class I genes, chemokine ligands and chemokine receptors. Effect sizes are reported as tumour and adjacent medians, with raw *p*-values and BH-adjusted q-values in Supplementary Table S3.

## 3. Results

### 3.1. Integrated Single-Cell Atlas Delineates Epithelial, Stromal and Immune Populations in PDAC

**Figure 1:**
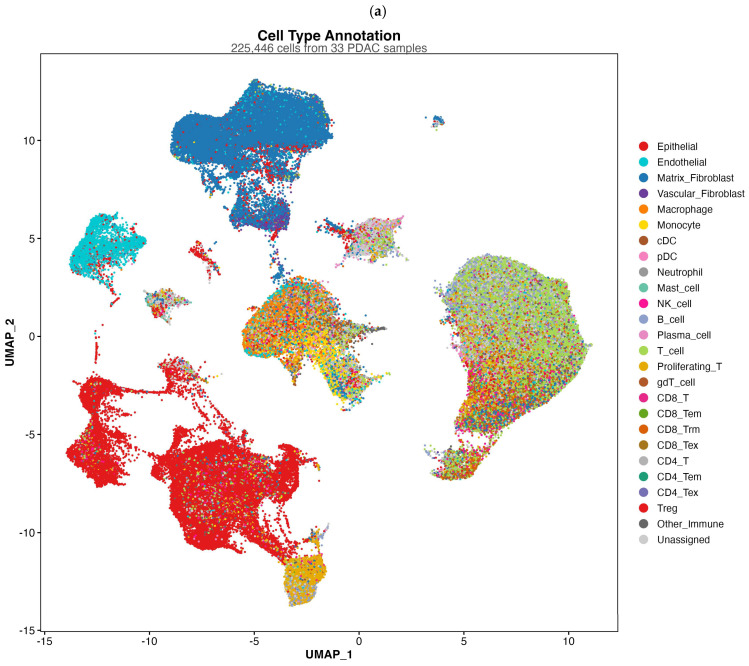

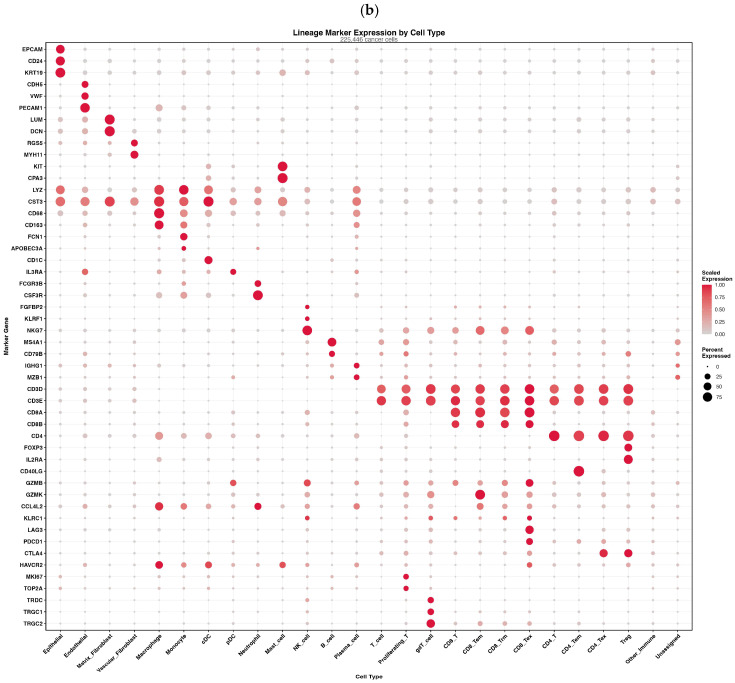
Cell type annotation of PDAC TME from single-cell RNA-seq datasets. (**a**). Integrated pancreatic ductal adenocarcinoma (PDAC) single-cell atlas and cell-type annotation. UMAP projection of 225,446 cells from 33 PDAC tumour samples only following QC, batch correction and integration. Cells are coloured using curated cell-type labels spanning epithelial, stromal, and immune compartments, including a discrete γδ T-cell population. (**b**). Canonical lineage marker expression validates cell-type annotation. Dot plot showing average log-normalised expression (colour intensity) and proportion of cells expressing each marker (dot size) across the annotated cell types. Markers include *EPCAM/KRT19* (epithelial), *COL1A1/DCN* (fibroblast), *PECAM1/VWF* (endothelial), *LST1/LYZ* (myeloid), *NKG7/FCGR3A* (NK), *MS4A1/CD79A* (B-cell), *MZB1/SDC1* (plasma), *CD3D/CD3E/TRAC* (T-cell) and *TRDC/TRGC1/TRGC2* (γδ T-cell).

An integrated analysis of publicly available PDAC single-cell transcriptomes identified 250,469 high-quality cells across 39 samples (33 PDAC tumours and 6 adjacent normal pancreatic tissue samples), enabling annotation of major epithelial, stromal, endothelial and immune compartments (Figure 1). The TME contained abundant malignant epithelial cells and fibroblast-rich stroma, alongside diverse myeloid and lymphoid populations, including monocytes/macrophages, dendritic cells, neutrophils and mast cells, as well as NK, B, plasma and multiple T-cell states.

Lineage identity was supported by canonical marker expression patterns (Figure 1), with epithelial (*EPCAM*, *KRT8/18/19*), endothelial (*PECAM1, VWF*) and stromal genes (*DCN*, *LUM*, *COL1A1*) validating non-immune compartments, and immune markers (*PTPRC*, *LYZ*, *FCGR3A*, *NKG7*, *CD3D/E*, *MS4A1*, *MZB1*) confirming myeloid and lymphoid subsets. Within the T-cell compartment, distinct transcriptional states were evident, including proliferating T-cells (*MKI67*, *TOP2A*), regulatory T-cells (*FOXP3*, *IL2RA*), and exhausted phenotypes (*PDCD1*, *LAG3*, *HAVCR2*).

### 3.2. γδ T-Cells Infiltrate PDAC Tumours with Marked Inter-Sample Heterogeneity

**Figure 2:**
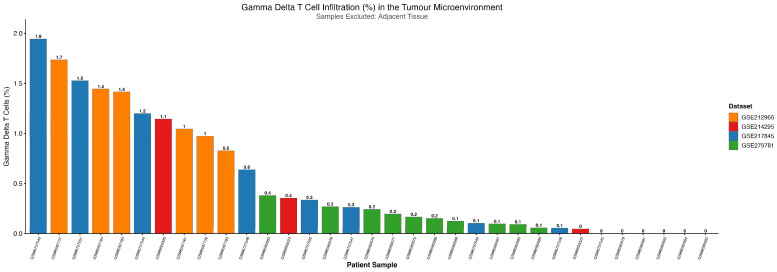
γδ T-cell infiltration in the pancreatic cancer tumour microenvironment. Bar plot shows the proportion of γδ T-cells among all cells in each tumour sample, ordered from highest to lowest infiltration; adjacent normal tissue samples were excluded. Bars are colour-coded by GEO series (GSE212966, GSE214295, GSE217845 and GSE279781), illustrating inter-patient and inter-dataset variability in γδ T-cell infiltration across pancreatic ductal adenocarcinoma samples.

A discrete γδ T-cell population was detected within the immune compartment, characterised by expression of *TRDC* and *TRGC1/TRGC2* alongside pan–T-cell genes (*CD3D/E*) (Figure 1). Quantification of γδ T-cell infiltration across tumour samples (n = 33) demonstrated substantial inter-sample variation (Figure 2). In most tumours, γδ T-cells constituted a minor fraction of all cells (<0.5%), whereas a subset of samples showed clear enrichment, reaching approximately 1–2% of total cells. These data indicate that γδ T-cell infiltration is present but unevenly distributed across PDAC tumours, consistent with patient-to-patient differences in immune composition.

### 3.3. Regulatory Molecule Profile of PDAC-Infiltrating γδ T-Cells Identifies Selective Modulation of KLRC1/NKG2A

**Figure 3:**
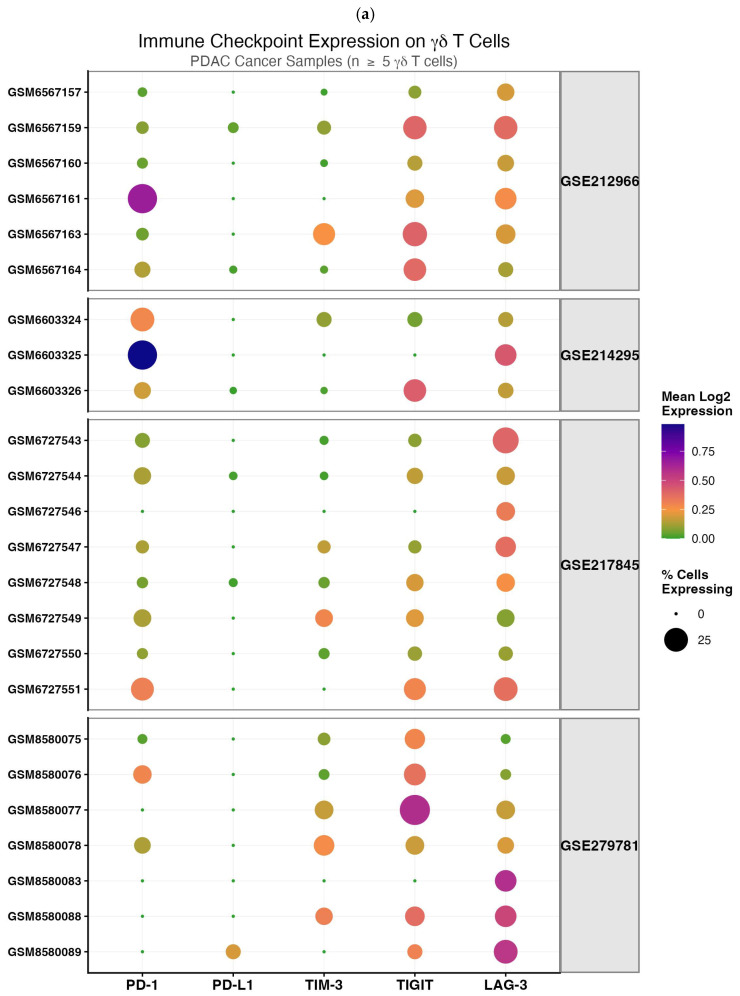

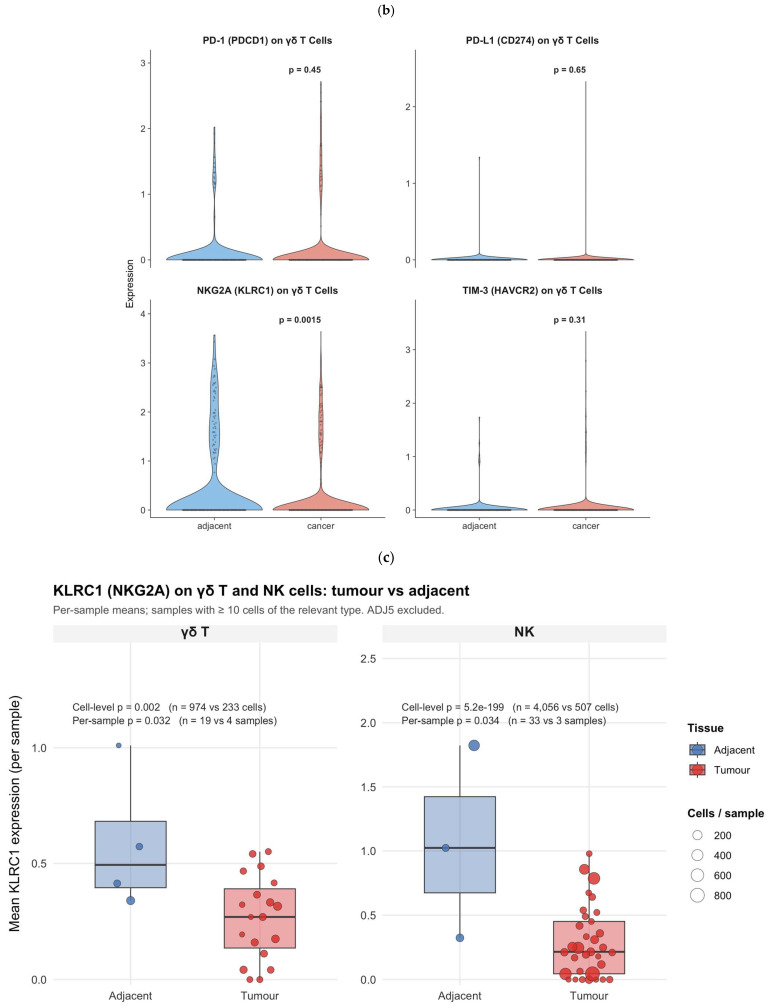
Immune checkpoint expression in γδ T-cells and *KLRC1*/NKG2A comparison in γδ-T and NK cells. (**a**) Dot plot showing expression of selected checkpoint genes in tumour-infiltrating γδ T-cells across individual PDAC samples, grouped by GEO cohort. Dot colour indicates mean log-normalised expression, and dot size indicates the percentage of γδ T-cells expressing each gene. (**b**) Tumour versus adjacent comparison of *PDCD1*/PD-1, *CD274*/PD-L1, *KLRC1*/NKG2A and *HAVCR2*/TIM-3 expression in γδ T-cells. (**c**) Sample-level mean KLRC1/NKG2A expression in γδ T-cells and NK cells from adjacent normal and tumour samples. Only samples with ≥10 cells in the relevant compartment were included, and ADJ5 was excluded. Each point represents one sample, with point size proportional to the number of cells contributing to the sample-level mean. *p*-values shown are from two-sided Wilcoxon rank-sum tests. Panel (**a**) includes tumour samples with ≥5 gated γδ T-cells.

To characterise inhibitory signalling pathways relevant to γδ T-cell function, expression of key checkpoint molecules was examined in γδ T-cells from tumour and adjacent normal tissue (Figure 3a). For γδ T-cell-specific sample-level visualisations, only samples with sufficient gated γδ T-cells were included; applying a ≥5 γδ T-cell threshold retained 24 of 33 tumour samples and 5 of 6 adjacent normal samples (Supplementary Table S2). Because only six adjacent normal samples were available, tumour versus adjacent comparisons should be interpreted as supportive and exploratory rather than definitive. PD-1 (*PDCD1*), PD-L1 (*CD274*) and TIM-3 (*HAVCR2*) were detectable in a subset of γδ T-cells, but their expression distributions did not differ significantly between tumour and adjacent normal tissue in the cell-level analysis shown in Figure 3b (Wilcoxon *p* = 0.45, 0.65 and 0.31, respectively; BH-q > 0.05 within the checkpoint family). In contrast, *KLRC1*/NKG2A expression in γδ T-cells differed between adjacent normal and tumour samples at the single-cell level (*p* = 0.0015, BH-q < 0.01; Figure 3b), consistent with prior evidence that NKG2A can function as an inhibitory receptor on cytotoxic lymphocytes [23,24,46]. In sample-level summaries, *KLRC1*/NKG2A expression showed the same directional pattern, with lower median expression in tumour-associated γδ T-cells than in adjacent normal γδ T-cells, although this comparison did not remain significant after family-wise correction (per-sample median tumour 0.319 vs. adjacent 0.414; raw *p* = 0.078, BH-q = 0.361; Supplementary Table S3). The per-sample *p* = 0.032 shown in Figure 3c is an unadjusted value from a stricter subset analysis (≥10 γδ T-cells, ADJ5 excluded; 19 tumour vs. 4 adjacent) and does not change this corrected, non-significant conclusion. These findings support *KLRC1*/NKG2A as an exploratory transcriptomic signal rather than definitive evidence of functional inhibition. Because NKG2A is also a dominant inhibitory receptor on NK cells, and because the HLA-E–NKG2A/CD94 pathway is classically described in NK-cell regulation, NK cells were included as a biologically relevant comparator [20,22,23,24,46,47] (Figure 3c). In tumour samples, *KLRC1*/NKG2A expression in γδ T-cells and NK cells showed overlapping patterns, suggesting that this candidate inhibitory programme may represent a broader cytotoxic-lymphocyte regulatory feature within the PDAC microenvironment.

Orthogonal validation using the Song et al. TCR module-score approach [42] showed concordant directionality for *KLRC1*/NKG2A, with lower tumour-associated γδ T-cell *KLRC1* under both the primary strict gate and the alternative TCR module-score gate (Supplementary Figures S1–S3).

### 3.4. Tumour Epithelial Cells Show Increased HLA-E Expression, Supporting a Potential HLA-E–NKG2A Regulatory Axis

**Figure 4:**
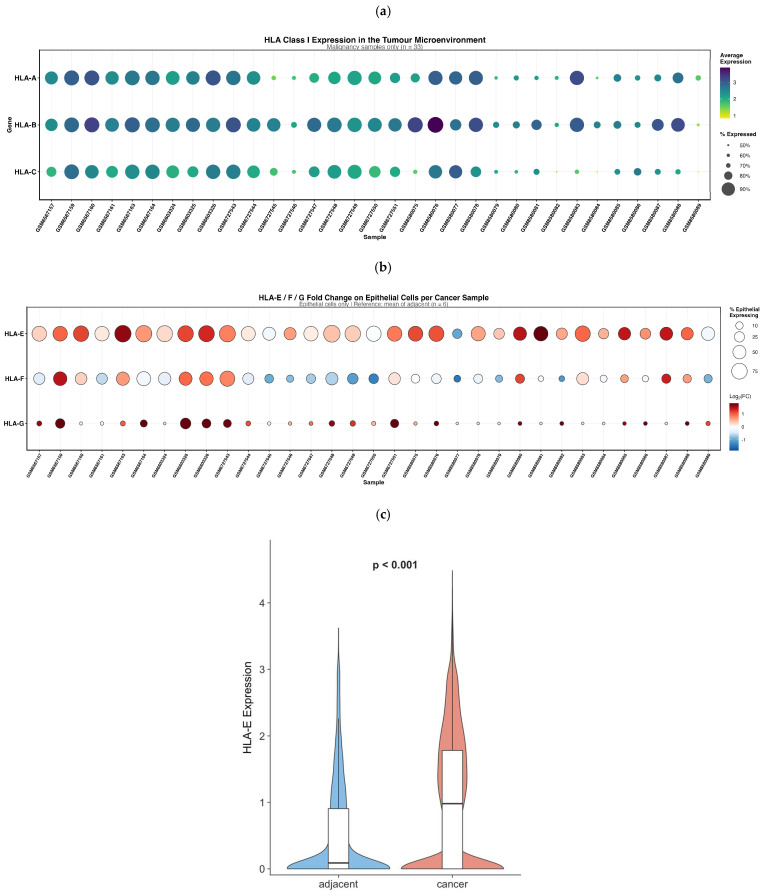
Classical and non-classical HLA class I expression in epithelial cells from PDAC and adjacent normal samples. (**a**) Bubble plot showing per-sample expression of classical HLA class I genes, HLA-A, HLA-B and HLA-C, across tumour samples. Dot colour indicates mean expression, and dot size indicates the percentage of cells expressing each gene. (**b**) Per-sample fold change in epithelial HLA-E, HLA-F and HLA-G expression in PDAC samples relative to the mean expression in adjacent normal epithelial cells. Dot colour indicates fold change, and dot size indicates the percentage of epithelial cells expressing each gene within each sample. (**c**) Cell-level epithelial HLA-E expression in tumour versus adjacent normal samples. Corresponding sample-level summaries are provided in Table 1 and Supplementary Table S3. *p*-values are from Wilcoxon rank-sum tests, as labelled.

HLA class I antigen-presenting machinery was preserved across tumour samples, with broad expression of HLA-A, HLA-B and HLA-C in both epithelial and immune compartments. However, HLA class I expression was found to be reduced in some samples within the TME (Figure 4a).

HLA-E, a non-classical MHC class I molecule that interacts with the inhibitory CD94/NKG2A receptor, was detected across multiple cell types and showed higher epithelial expression in tumour than adjacent tissue in sample-level summaries (median 1.06 vs. 0.57; raw *p* = 0.012, BH-q = 0.035), consistent with increased transcriptomic availability of HLA-E in PDAC (Figure 4b; Supplementary Table S3) [23,24,46,47]. Focusing on epithelial cells, HLA-E expression was quantified per sample across 33 PDAC tumours and 6 adjacent normal pancreatic tissue samples (Table 1; adjacent median 0.617). The proportion of HLA-E-positive epithelial cells was variable in tumours (median 67.86%; IQR 50.56–77.85; range 18.54–98.46%) and more constrained in adjacent tissue (median 55.90%; IQR 51.80–61.40; range 41.95–73.91%). HLA-G showed inter-sample heterogeneity but was not pursued further in the present analysis.

To assess whether these observations were driven by a single public cohort, we performed dataset-stratified visualisation of the key findings. γδ T-cell abundance, γδ T-cell *KLRC1*/NKG2A expression and epithelial HLA-E expression varied across GEO cohorts, but the overall patterns were not attributable to one dataset alone (Supplementary Figures S4–S6). These analyses support the robustness of the integrated findings while also highlighting inter-cohort heterogeneity.

To assess whether the epithelial HLA-E signal reflected malignant epithelial populations, we performed inferCNV-based malignant epithelial classification. Tumour samples were enriched for CNV-positive epithelial cells, and HLA-E expression remained directionally higher when restricted to CNV-confirmed malignant tumour epithelium compared with CNV-negative adjacent epithelium (all cohorts *p* = 0.062; within GSE212966 *p* = 0.030; Supplementary Tables S5 and S6).

Ligand–receptor inference using LIANA and CellChat provided additional transcriptomic support for HLA-E–*KLRC1/KLRD1* as a candidate cytotoxic-lymphocyte interaction in PDAC. In LIANA, tumour γδ T-cells were predicted to receive HLA-E-mediated signals mainly from immune-cell sources, including γδ T-cells themselves (aggregate rank = 0.110), CD4 T-cells (0.125), CD8 T-cells (0.132), macrophages (0.181) and NK cells (0.228), whereas epithelial-to-γδ T-cell interactions ranked at the floor of the output (aggregate rank = 1.000). In adjacent tissue, NK cells and CD8 T-cells were the leading predicted HLA-E sources to γδ T-cells (aggregate ranks 0.262 and 0.294, respectively). CellChat detected HLA-E→*KLRC1* communication only in adjacent tissue, with NK cells as the dominant predicted receivers (NK→NK probability 0.102, CD8_Trm→NK 0.089, CD8_Tex→NK 0.089, γδ T→NK 0.085). In tumour tissue, HLA-E→*KLRC1* was absent from CellChat’s overexpression-filtered output, in keeping with lower *KLRC1* expression in tumour NK cells (log2FC −2.25, cell q < 0.001) and γδ T-cells (log2FC −0.71, cell q = 0.003; Supplementary Table S4). Underlying expression supported these inferences: HLA-E was expressed by 60.9% of tumour epithelial cells and 50.7% of adjacent epithelial cells, whereas Table 1 reports per-sample median HLA-E positivity of 67.86% and 55.90%, respectively. *KLRC1* was expressed by 14.7% of tumour γδ T-cells and 22.9% of adjacent γδ T-cells. These findings support the candidacy of the HLA-E–NKG2A axis as a cytotoxic-lymphocyte regulatory pathway within the PDAC tumour microenvironment.

We also examined antigen-presentation machinery in epithelial cells. B2M, TAP1, TAP2 and classical HLA class I genes showed coordinated tumour-associated expression, while *KLRD1*/CD94 was not expressed in epithelial cells, as expected (Supplementary Figure S8). These findings support the transcriptomic availability of the MHC class I antigen-presentation pathway, but do not prove surface HLA-E protein expression or functional receptor engagement.

### 3.5. Chemokine Landscape Reveals CCL5/CCL4- and CXCL8-Rich Niches and Candidate Trafficking Receptor Programmes in γδ T-Cells

**Figure 5:**
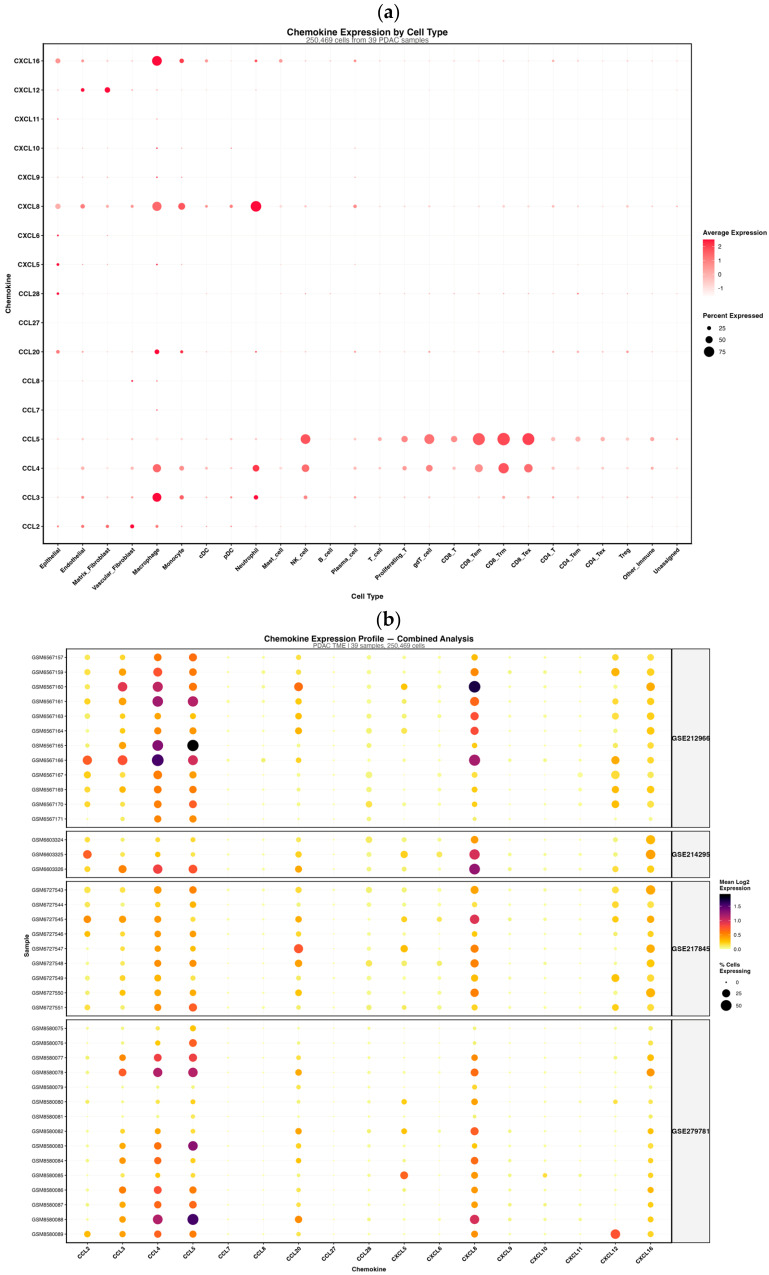

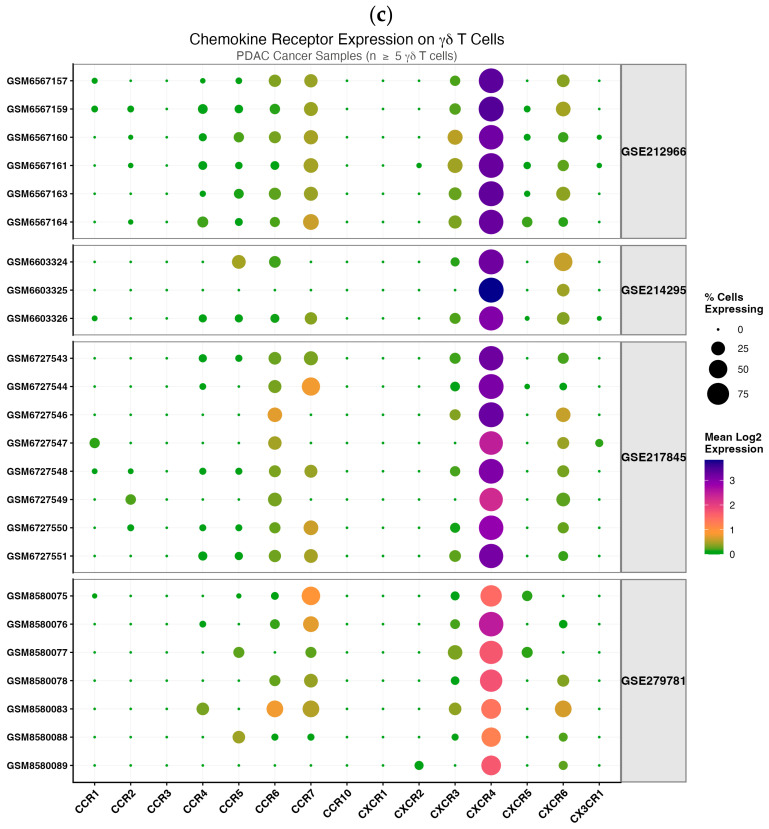
Chemokine ligand and receptor landscape in the PDAC microenvironment. (**a**) Dot plot showing selected chemokine ligands across annotated cell populations. Dot colour indicates mean log-normalised expression, and dot size indicates the percentage of cells expressing each ligand within the indicated cell type. (**b**) Sample-level chemokine ligand expression across tumour and adjacent normal samples, grouped by GEO cohort. Dot colour indicates mean log-normalised expression, and dot size indicates the percentage of all cells within each sample expressing the ligand. (**c**) Chemokine receptor expression in γδ T-cells across individual PDAC samples. Dot colour indicates mean log-normalised expression among γδ T-cells, and dot size indicates the percentage of γδ T-cells expressing each receptor. Interferon-inducible CXCL9/10/11 signals were sparse, with ≤1.03% of all cells positive across the integrated atlas. Panel (**c**) includes samples with ≥5 gated γδ T-cells; whole-atlas chemokine ligand analyses in panel (**b**) used all 39 samples, comprising 33 tumour samples and 6 adjacent normal samples.

Chemokine ligand expression across the integrated atlas highlighted a microenvironment enriched for myeloid- and fibroblast-associated inflammatory signals. Across cell types, CCL2 and CCL7 were prominent within myeloid populations, while CXCL8 was enriched in macrophages, and CXCL12/CXCL14 were preferentially expressed by stromal fibroblast states (Figure 5a). Sample-level profiling showed substantial inter-patient heterogeneity in chemokine programmes, with the highest chemokine burdens in macrophage-rich tumours (Figure 5b). Notably, interferon-inducible T-cell-attracting ligands CXCL9, CXCL10 and CXCL11 were not broadly dominant in many samples.

Signals include myeloid-derived CCL2/CCL3/CCL4/CCL7/CXCL8, stromal CXCL12/CXCL14, and epithelial-associated CXCL16, outlining potential recruitment cues for cytotoxic lymphocytes, including γδ T-cells. Within tumour-infiltrating γδ T-cells, CXCR4 showed the strongest receptor expression, followed by CCR6, CCR7 and CXCR6, with marked inter-sample heterogeneity (Figure 5c, Supplementary Figure S7). These patterns suggest that PDAC-associated γδ T-cells may engage candidate chemokine receptor programmes within tumour tissue, although the current analysis does not directly demonstrate chemokine-driven positioning.

## 4. Discussion

Our integrated analysis of PDAC scRNA-seq datasets provides a transcriptome-level map of tumour-infiltrating γδ T-cells and highlights candidate regulatory pathways that may shape their function in the pancreatic TME. Across 39 samples (33 tumours and 6 adjacent normal tissues), γδ T-cells were detectable but highly variable in abundance, consistent with the broader inter-patient heterogeneity that characterises PDAC immune landscapes.

Prior work has suggested that γδ T-cells can constitute a substantial fraction of tumour-infiltrating T-cells in PDAC and may restrain αβ T-cell immunity through checkpoint ligands such as PD-L1 and Galectin-9 [14]. In contrast, studies of treatment-naïve patients and complementary in silico analyses describe marked variability and, in some cohorts, lower frequencies of tumour-infiltrating γδ T-cells with evidence of tumour microenvironment-driven functional reprogramming [48]. Our findings support this heterogeneity: most tumours showed low γδ T-cell proportions, while a subset showed enrichment. This variation should not be interpreted solely as a technical effect. Although cohort composition, tissue sampling and dissociation may contribute to measured γδ T-cell frequency, γδ-enriched and γδ-sparse tumours may also reflect genuine biological heterogeneity. Larger clinically annotated cohorts will be required to determine whether γδ T-cell-enriched tumours represent a distinct clinical or transcriptomic subgroup.

Reduced expression of classical HLA I molecules (HLA A/B/C) in some samples limits αβ T-cell mediated cellular cytotoxicity. This may serve as an immune evasion mechanism in pancreatic cancer [49]. HLA-G expression was visualised for context but not interpreted further, as it was not a primary focus of the present analysis. Most samples exhibited high HLA-E expression. Elevated levels of HLA-E and HLA-G have been independently linked to poor prognosis in pancreatic cancer [50]. Beyond abundance, our analysis prioritised regulatory molecules that could mediate immune suppression. We observed differential expression of *KLRC1* (NKG2A) in γδ T-cells between tumour and adjacent tissue, and importantly, increased epithelial expression of the NKG2A ligand HLA-E in tumours. Together, these transcriptomic findings nominate HLA-E–NKG2A as a plausible candidate inhibitory pathway in PDAC, although surface protein expression, spatial proximity and functional suppression were not directly measured. This axis is mechanistically attractive because it links tumour or stromal ligand availability with NKG2A-expressing cytotoxic immune cells, including γδ T-cells and NK cells, potentially contributing to dampened anti-tumour activity in an already suppressive microenvironment.

Ligand-receptor inference using CellChat [44] and LIANA [45] provided additional hypothesis-generating support for HLA-E–*KLRC1/KLRD1* communication in the PDAC microenvironment. These analyses suggested that NK cells were the dominant predicted receivers of HLA-E-mediated inhibitory signalling, while γδ T-cells showed lower-level predicted interactions. We therefore interpret HLA-E–NKG2A as a broader cytotoxic-lymphocyte regulatory axis rather than a γδ T-cell-exclusive mechanism.

Chemokine analyses suggested that PDAC-associated γδ T-cells exist within a heterogeneous trafficking milieu, with variable expression of CXCR4, CCR6, CCR7 and CXCR6. The ligand profile was consistent with key features of the PDAC microenvironment, including myeloid-associated CCL2/CCL4/CCL5 and CXCL8 signals, together with stromal CXCL12 enrichment. These cues may favour myeloid recruitment or retention and shape lymphocyte localisation within desmoplastic tumour niches. However, these findings do not directly demonstrate chemokine-driven positioning; rather, they identify candidate ligand-receptor programmes that could be tested in spatial or functional models and may inform future γδ T-cell-based therapeutic strategies. Comparable heterogeneity in chemokine programmes linked to γδ T-cell recruitment has been reported in biliary tract cancer, supporting the concept that context-specific chemokine regulation might shape γδ T-cell infiltration across hepatopancreatobiliary tumours [51].

These findings also intersect with emerging adoptive and engineered cell strategies. A report of NLRP3 pathway stimulation described expansion of tumour-reactive TCR γδ+ TIL and CD1d-restricted recognition of PDAC cells, accompanied by induction of CXCL9/10 [52]. Although baseline CXCL9/10 signals were limited in our datasets, the observation that these chemokines can be induced ex vivo suggests a potential avenue to enhance tissue invasion and functional engagement of γδ T-cell products, particularly if inhibitory checkpoints such as NKG2A are simultaneously addressed.

Key limitations include reliance on publicly available datasets with variable tissue processing, sequencing depth, sample handling and annotation; a relatively small set of adjacent normal samples; and the use of transcript abundance as a proxy for surface protein expression. Although we performed sample-level summaries, dataset-stratified analyses and inferCNV-based epithelial-cell sensitivity analyses, tumour–adjacent comparisons may still be influenced by patient-level, cohort-level and batch-related effects despite Harmony integration. Dedicated doublet-calling tools were not uniformly applied across all public datasets, so residual doublet contamination cannot be excluded. In addition, ligand–receptor inference and antigen-presentation analyses provide transcriptomic support for a candidate HLA-E–NKG2A axis but do not prove surface protein expression, spatial proximity or functional inhibition. Prospective validation using paired tumour and adjacent samples, spatial profiling, orthogonal protein measurements and functional assays will be required to confirm the HLA-E–NKG2A interaction and define its consequences for γδ T-cell and NK-cell activity in PDAC.

## 5. Conclusions

In summary, integrated scRNA-seq analysis across 33 PDAC tumours and 6 adjacent normal samples identifies heterogeneous γδ T-cell infiltration and nominates HLA-E–NKG2A as a candidate regulatory axis within the PDAC microenvironment. The inclusion of NK cells as a comparator supports the biological relevance of this pathway beyond γδ T-cells, while chemokine receptor programmes suggest candidate trafficking features that may shape γδ T-cell localisation. These hypotheses provide a rationale for targeted mechanistic studies using spatial, protein-level and functional validation.

## Figures and Tables

**Table 1:** HLA-E expression in epithelial cells by tissue category (per-sample summaries).

Tissue	Samples (n)	Epithelial Cells (n)	HLA-E+ Cells, Median (IQR) (%)	Range (%)	Mean Expression, Median (IQR)	Range (Mean Expr)
Adjacent	6	3533	55.90 (51.80–61.40)	41.95–73.91	0.617 (0.509–0.838)	0.310–1.052
Tumour	33	59,831	67.86 (50.56–77.85)	18.54–98.46	1.070 (0.787–1.275)	0.248–2.024

## Data Availability

scRNA-seq datasets analysed in this study are publicly available from the NCBI Gene Expression Omnibus under accession numbers GSE212966, GSE214295, GSE217845 and GSE279781.

## Supplementary Materials

The following supporting information can be downloaded at: https://www.mdpi.com/article/10.3390/cancers18111723/s1, Figure S1: γδ T-cell gating, TCR module score quadrant; Figure S2: γδ T-cell marker validation across gates; Figure S3: KLRC1 sensitivity to γδ T-cell gating definition; Figure S4: γδ T-cell abundance by cohort; Figure S5: *KLRC1* cross-cohort consistency; Figure S6: HLA-E cross-cohort consistency; Figure S7: Chemokine receptors by cohort; Figure S8: Antigen-presentation machinery in epithelium. Table S1: γδ T-cell gating threshold sensitivity; Table S2: Per-sample γδ T-cell counts; Table S3: Family-wise Benjamini–Hochberg corrected *p*-values; Table S4: Master statistics table, BH-corrected gene family; Table S5: Per-sample malignant epithelial cell classification (inferCNV); Table S6: HLA-E in CNV-confirmed malignant vs adjacent-normal epithelial cells; Table S7: Per-sample dataset, tissue, and post-QC cell counts.
