# Supplementary material for: Integrated Single-Cell Transcriptomics Identifies γδ T-Cell Heterogeneity and a Candidate HLA-E–NKG2A Regulatory Axis in Pancreatic Ductal Adenocarcinoma
Source: Cancers (Basel). 2026 May 25;18(11):1723. doi: 10.3390/cancers18111723 (PMC13255768; doi:10.3390/cancers18111723)
Supplement: Supplementary file 1 [file cancers-18-01723-s001.zip › cancers-4292279-supplementary.pdf]

Supplementary Materials

Integrated Single-Cell Transcriptomics Identifies γδ T-Cell Heterogeneity and a Candidate Cytotoxic-Lymphocyte HLA-E–NKG2A Regulatory Axis in Pancreatic Ductal Adenocarcinoma

Contents

Supplementary Methods

M0: Data sources, pre-processing, and quality control

M1: γδ T-cell identity validation

M2: Malignant epithelial cell classification (inferCNV)

M3: Dataset stratification

M4: Antigen-presentation machinery analysis

M5: Ligand-receptor inference (CellChat and LIANA)

M6: Family-wise multiple-testing correction

Supplementary Figures S1–S8

Figure S1: γδ T-cell gating, TCR module score quadrant

Figure S2: γδ T-cell marker validation across gates

Figure S3: KLRC1 sensitivity to γδ T-cell gating definition

Figure S4: γδ T-cell abundance by cohort

Figure S5: KLRC1 cross-cohort consistency

Figure S6: HLA-E cross-cohort consistency

Figure S7: Chemokine receptors by cohort

Figure S8: Antigen-presentation machinery in epithelium

Supplementary Tables S1–S7

Table S1: γδ T-cell gating threshold sensitivity

Table S2: Per-sample γδ T-cell counts

Table S3: Family-wise Benjamini–Hochberg corrected p-values

Table S4: Master statistics table, BH-corrected gene family

Table S5: Per-sample malignant epithelial cell classification (inferCNV)

Table S6: HLA-E in CNV-confirmed malignant vs adjacent-normal epithelial cells

Table S7: Per-sample dataset, tissue, and post-QC cell counts

Supplementary Methods

M0: Data sources, pre-processing, and quality control

Single-cell RNA-seq data from four publicly available human pancreatic ductal adenocarcinoma (PDAC) cohorts were obtained from the Gene Expression Omnibus (GEO): GSE212966 (Chen et al., J Transl Med 2023;21:210) [32] - 12 samples comprising 6 PDAC tumour and 6 matched adjacent non-tumour tissues sequenced on Illumina NovaSeq 6000 from treatment-naïve patients at Peking University First Hospital; GSE214295 (Chen et al., Cancer Lett 2024;576:216586) [35] - 3 primary PDAC samples profiled alongside paired organoids; GSE217845 (Caronni et al., Nature 2023;623:415-422) [33] - 9 PDAC tumour samples; and GSE279781 (Montagne et al., iScience 2025;28:111569) [34] - 15 samples from a neoadjuvant clinical trial (NCT02451982; GVAX vaccine alone, with anti-PD-1, or with anti-PD-1 + CD137 agonist). All cohorts used the 10X Genomics Chromium single-cell platform. Per-sample metadata, cohort assignment, tissue type, and post-QC cell counts are provided in Supplementary Table S7.

The filtered cell-by-gene count matrices distributed by each GEO submission served as input. Quality control was applied uniformly across all samples using Seurat: cells were retained if they expressed at least 200 genes (nFeature_RNA ≥ 200), had at least 500 transcripts (nCount_RNA ≥ 500), and had mitochondrial gene expression ≤ 20% (percent.mt ≤ 20%). Counts were log-normalised using Seurat's NormalizeData with default settings. Cohort batch effects were corrected using Harmony (Korsunsky et al., Nat Methods 2019;16:1289) [38], with the dataset variable as the integration covariate. After QC and integration, 250,469 cells from 39 samples (33 tumour + 6 adjacent) were retained for downstream analysis.

M1: γδ T-cell identity validation

Two TCR module scores were computed within the lymphoid compartment (99,536 cells) using Seurat's AddModuleScore (nbin = 24, ctrl = 100): a γδTCR score based on TRDC and an αβTCR score based on TRAC, TRBC1, TRBC2. Putative γδ T-cells under the Song gate were defined by γδTCR > 0.05 and αβTCR < 0. Reference: Song Z et al., J Leukoc Biol 2023;114:630–641 (PMID 37648670) [42].

M2: Malignant epithelial cell classification (inferCNV)

To distinguish CNV-positive malignant epithelial cells from CNV-negative non-malignant epithelium, copy-number inference was performed using inferCNV (Tickle T et al., Broad Institute) [43] on each sample's epithelial cluster, with immune and stromal cells from the same sample serving as the within-sample reference population. inferCNV was run sample-wise to avoid batch-effect contamination of the CNV reference. Cells with a CNV signal above the non-malignant reference distribution were classified as malignant; cells indistinguishable from the reference were classified as non-malignant. Classification was completed for 38 of 39 samples; one adjacent sample (GSM6567171_ADJ6) was not classified and is omitted from CNV-stratified analyses. Per-sample malignant cell counts and percentages are given in Supplementary Table S5. The HLA-E expression analysis was re-tested on the CNV-confirmed malignant subset

(Supplementary Table S6) to verify that the elevation in 'tumour epithelium' is driven by malignant cells specifically and not by non-malignant epithelial cells contaminating the tumour-sample epithelial cluster.

M3: Dataset stratification

Within-cohort tumour-vs-adjacent comparison was performed for GSE212966 (the only cohort with paired adjacent samples: 5 adjacent + 6 tumour). Cross-cohort consistency on tumour samples was assessed by the Kruskal-Wallis test across the four cohorts.

M4: Antigen-presentation machinery analysis

Per-gene Wilcoxon rank-sum tests at the cell level (all annotated epithelial cells) and per-sample level (samples with ≥ 10 epithelial cells; ADJ5 excluded). Eight genes: B2M, TAP1, TAP2, HLA-A, HLA-B, HLA-C, HLA-E, KLRD1. Benjamini–Hochberg correction across the 8-gene family. Cross-cohort Kruskal-Wallis on tumour samples to check cohort-driven effects. This analysis uses the annotated epithelial cluster (rather than the CNV-confirmed malignant subset specifically) so that the per-sample n is preserved for the 8 genes tested in parallel; the CNV-restricted HLA-E sensitivity test (Table S6) confirms the direction of the headline HLA-E finding when restricted to malignant cells only.

M5: Ligand–receptor inference

Two independent inference frameworks applied to a filtered cohort (samples with ≥ 10 γδ T cells: 19 tumour, 4 adjacent). CellChat (Jin et al., 2021) [44]: curated CellChatDB.human MHC-I subset, raw.use = TRUE, population.size = FALSE, min.cells = 10. LIANA (Dimitrov et al., 2022) [45]: consensus across NATMI, Connectome, SingleCellSignalR, logFC, and CellPhoneDB; OmniPath Consensus database. Both run separately on tumour and adjacent conditions. Note: LIANA represents NKG2A as the heterodimer KLRC1_KLRD1 (CD94/NKG2A).

M6: Family-wise multiple-testing correction

All primary tumour-vs-adjacent tests were assembled into a single statistical family (n = 19): antigen-presentation machinery (8), γδ T-cell checkpoint/receptor markers (6: PDCD1, CD274, KLRC1, HAVCR2, LAG3, TIGIT), NK cell KLRC1 (1), and γδ T-cell chemokine receptors within GSE212966 (4: CXCR4, CCR6, CCR7, CXCR6). Each was tested using the Wilcoxon rank-sum test at the cell-level and per-sample level (samples with ≥ 10 cells; ADJ5 excluded). Benjamini-Hochberg correction applied across the unified 19-test family separately for cell-level and per-sample p-values.

Supplementary Figures

Figures S1–S8 are provided on the following pages.

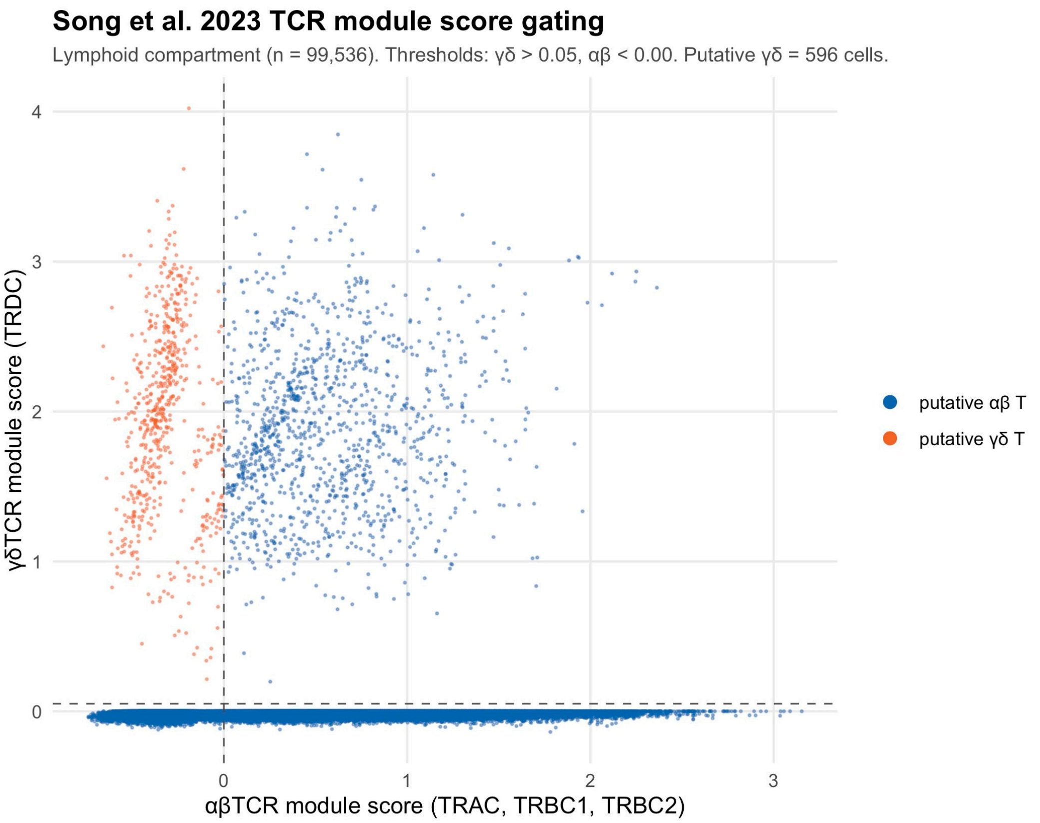

Figure S1: γδ T-cell gating by Song et al. 2023 TCR module score method.

Scatter plot of αβTCR module score (TRAC + TRBC1 + TRBC2) versus γδ TCR module score (TRDC) within the lymphoid compartment (n = 99,536 cells). Dashed lines indicate gating thresholds (γδ TCR > 0.05, αβTCR < 0). Putative γδ T-cells (orange, n = 596) occupy the upper-left quadrant; putative αβ T-cells (blue) occupy the right region.

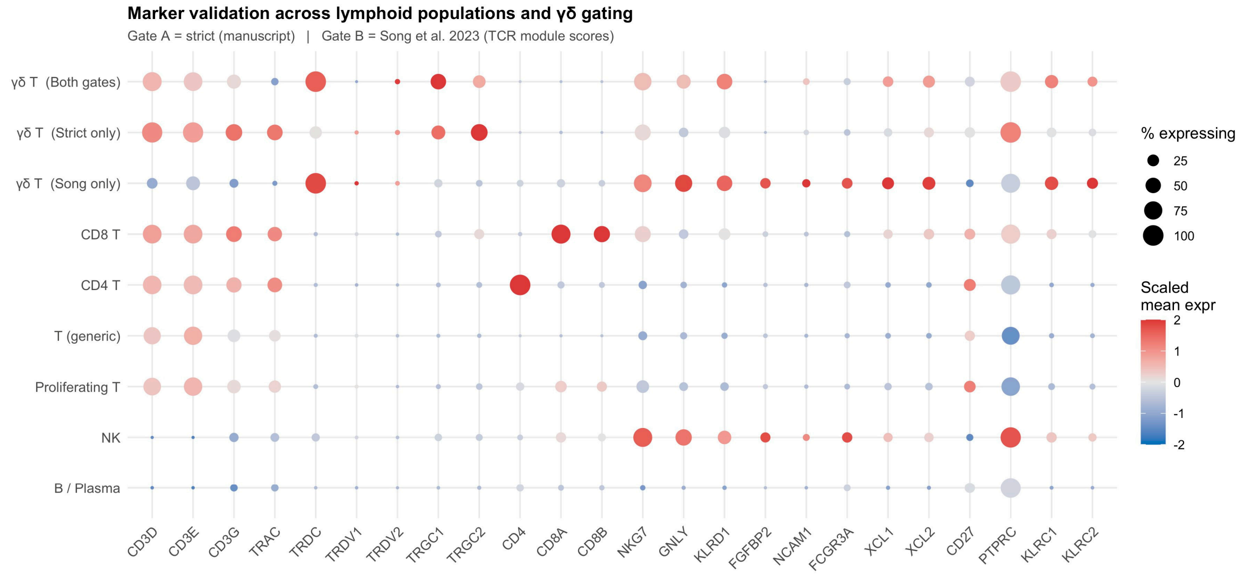

Figure S2: γδ T-cell marker validation across both gating definitions.

Dot size represents the percentage of cells expressing each marker; colour represents the scaled mean expression. The three γδ T-Cell rows show cells passing both gates (n = 86), strict-gate-only (n = 1,176), and Song-gate-only (n = 510). Cells captured uniquely by the Song et al. gating strategy show elevated TRDC, NK-like markers (NKG7, GNLY, KLRD1), and KLRC1, confirming the broader Song gate captures CD8α-expressing or NK-like γδ T-cells.

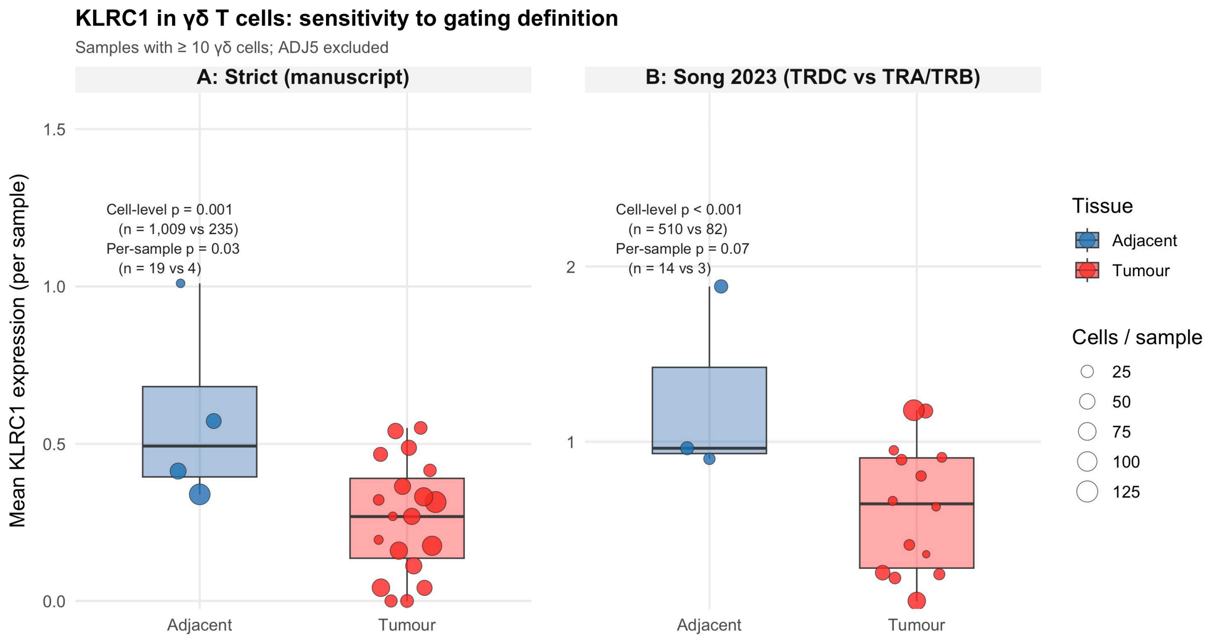

Figure S3: KLRC1 expression in γδ T-cells is robust to gating definition.

Per-sample mean KLRC1 expression (samples with ≥ 10 γδ T-cells; ADJ5 excluded). Both gates show concordant direction (decreased KLRC1 in tumour). Cell-level p-values are highly significant under both gates. Per-sample test is significant under the strict gate ($p = 0.03$) and borderline under the Song gate ($p = 0.07$) due to reduced adjacent-sample power (3 samples).

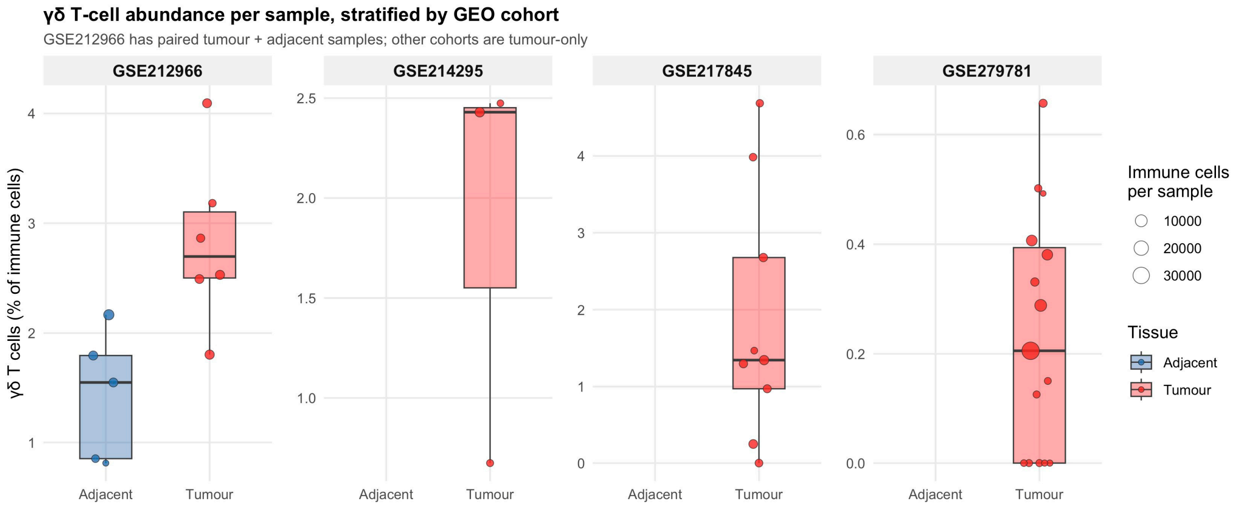

Figure S4: γδ T-cell abundance per sample, stratified by GEO cohort.

Within GSE212966 (paired cohort), tumour samples show higher γδ T-cell percentage of immune cells than adjacent. GSE279781 shows approximately 10-fold lower γδ T-Cell abundance than the other three cohorts; this is flagged as a caveat for chemokine interpretation in the discussion.

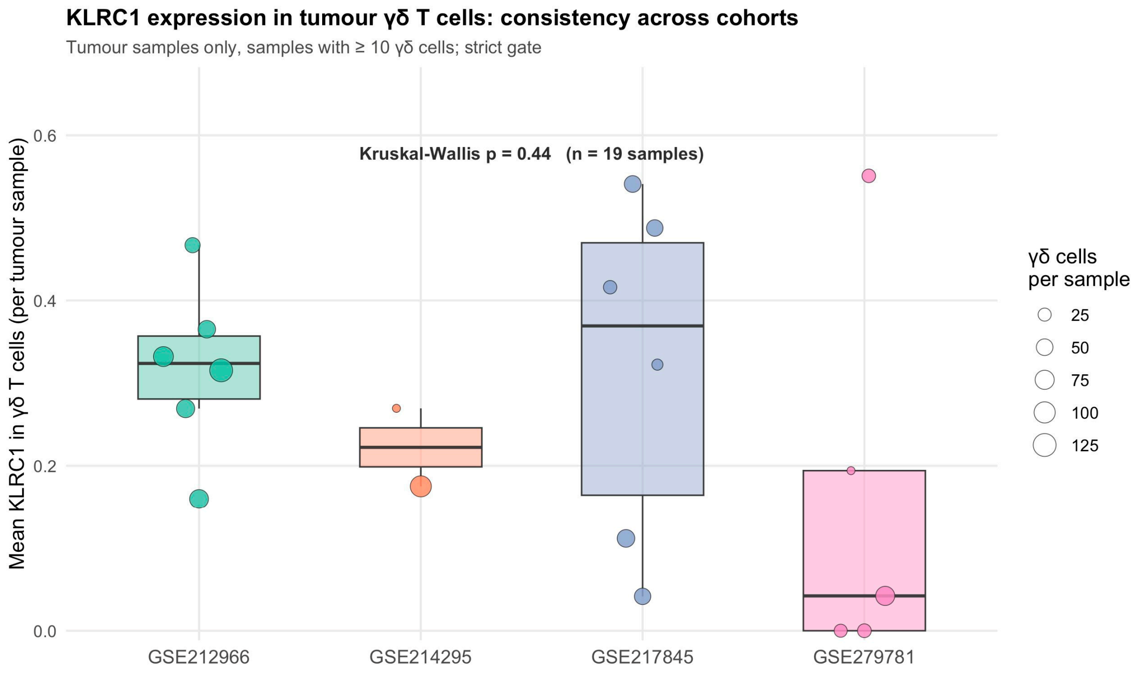

Figure S5: KLRC1 in tumour γδ T-cells is consistent across the four GEO cohorts.

Per-sample mean KLRC1 in tumour samples with ≥ 10 γδ T-cells. Kruskal-Wallis $p = 0.44$ indicates no significant difference between cohorts.

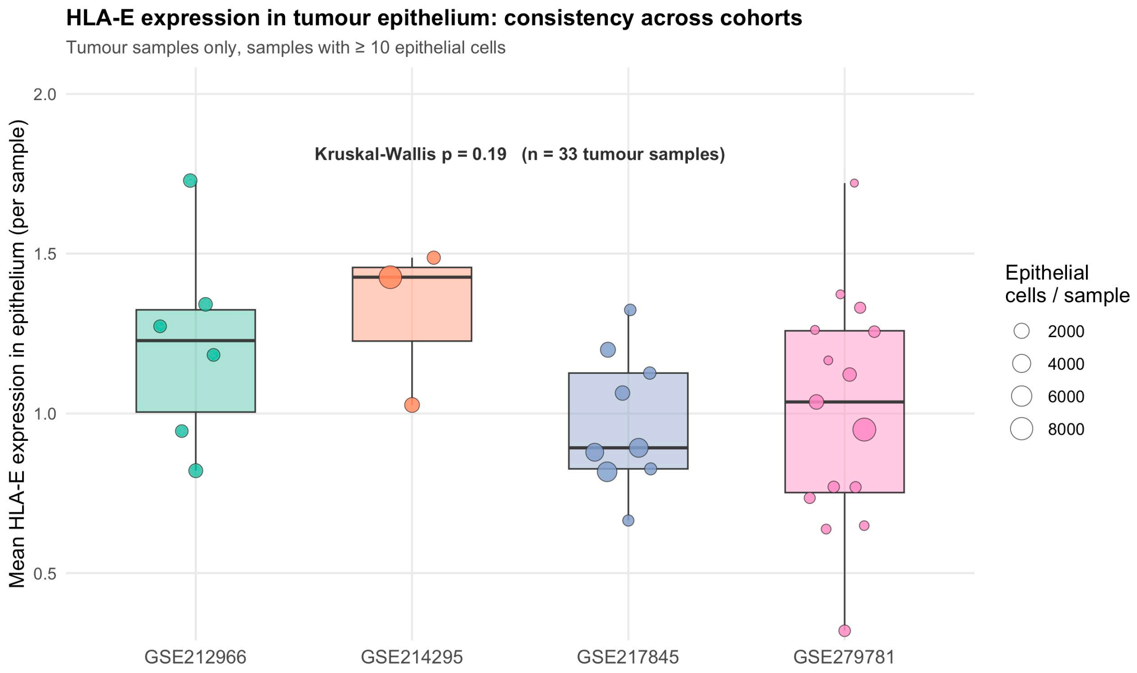

Figure S6: HLA-E in tumour epithelium is consistent across the four GEO cohorts.

Per-sample mean HLA-E in tumour samples with ≥ 10 epithelial cells (n = 33 samples). Kruskal-Wallis p = 0.19; epithelial HLA-E elevation is consistent across cohorts.

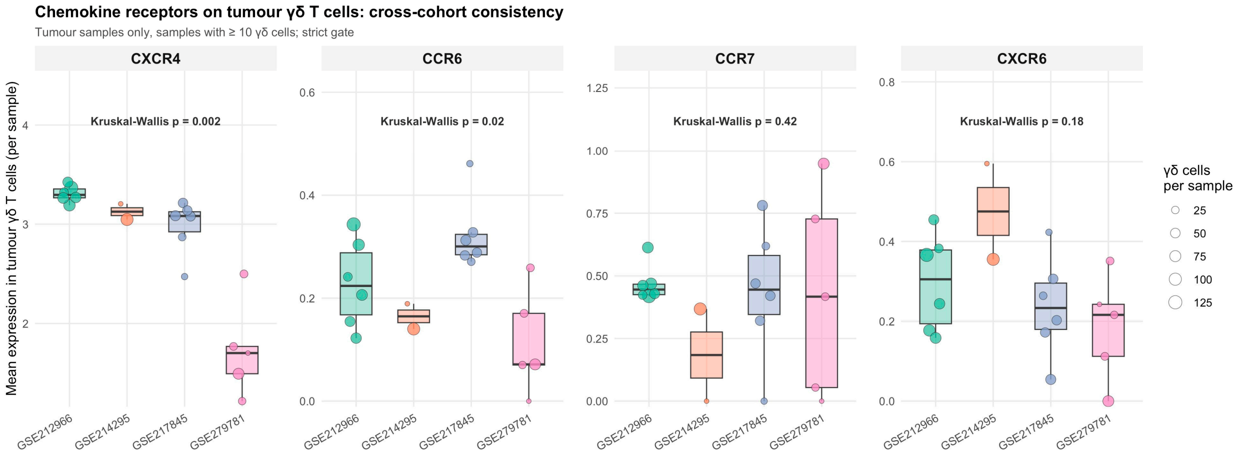

Figure S7: Chemokine receptor expression on tumour γδ T-cells across the four GEO cohorts.

CCR7 (Kruskal-Wallis $p = 0.42$) and CXCR6 ($p = 0.18$) are consistent across cohorts. CXCR4 ($p = 0.002$) and CCR6 ($p = 0.02$) show significant cohort variation, primarily driven by lower expression in GSE279781.

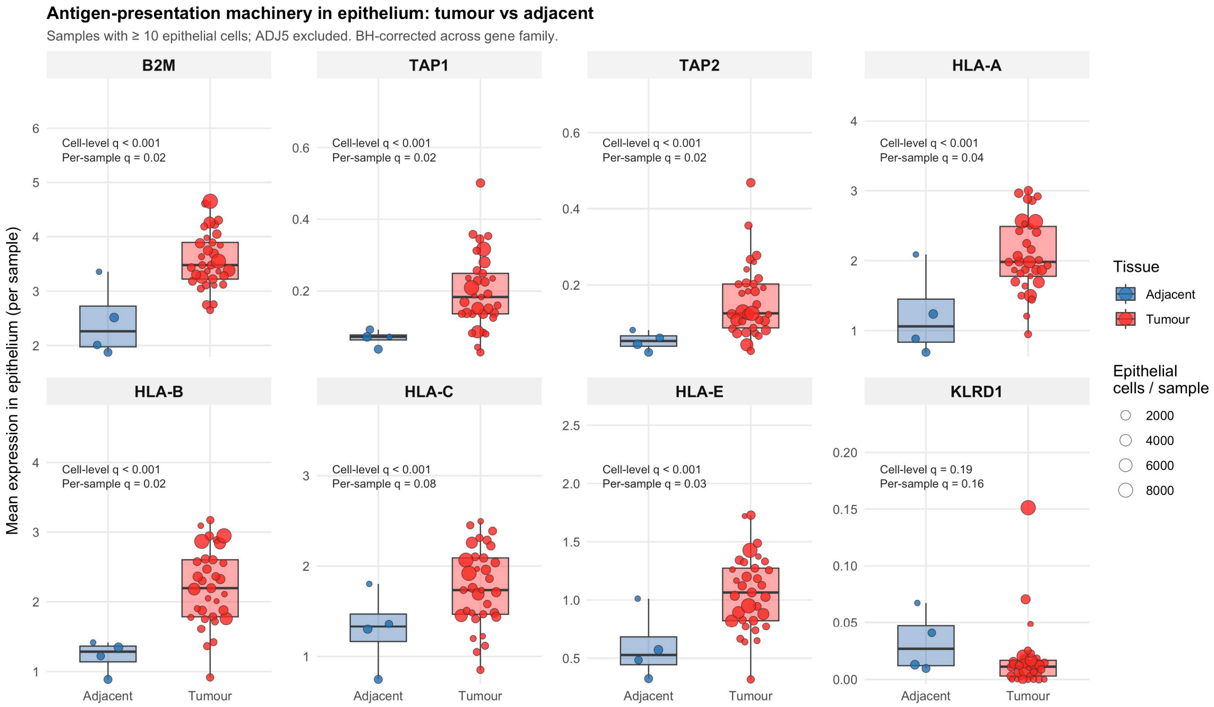

Figure S8: Antigen-presentation machinery in epithelium: tumour versus adjacent.

Eight-panel per-sample boxplots showing coordinated upregulation of B2M, TAP1, TAP2, HLA-A, HLA-B, HLA-C and HLA-E in tumour epithelium. KLRD1 (CD94) is appropriately not expressed by epithelium. q-values are Benjamini-Hochberg corrected across the 8-gene family. Cells are from the annotated epithelial cluster (per Methods M4); the CNV-restricted HLA-E sensitivity analysis is reported in Supplementary Table S6.

Supplementary Tables

Supplementary Table S1: γδ T-cell gating threshold sensitivity analysis.

Per-sample γδ T-cell abundance was recomputed under a grid of inclusion (0.3–0.7) and exclusion (0.03–0.10) threshold values. Spearman correlation of per-sample γδ T-cell abundance versus the primary threshold combination (inclusion 0.5, exclusion 0.05; highlighted) confirms that the gating result is robust to modest perturbations in cut-off choice. The Wilcoxon rank-sum p-value for KLRC1 expression in γδ T-cells (tumour vs adjacent) is shown for each condition; the rank-order of samples by γδ T-cell abundance is preserved ($\rho \geq 0.997$) across all conditions tested.

Inclusion threshold	Exclusion threshold (CD4/FGFBP2)	Spearman ρ vs primary	KLRC1 Wilcoxon p (γδ, tumour vs adjacent)
0.3	0.03	1.000	0.436
0.3	0.05	1.000	0.436
0.3	0.10	1.000	0.436
0.4	0.03	1.000	0.436
0.4	0.05	1.000	0.436
0.4	0.10	1.000	0.436
**0.5**	**0.05**	**1.000**	**0.436**
0.5	0.03	1.000	0.436
0.5	0.10	1.000	0.436
0.6	0.03	1.000	0.640
0.6	0.05	1.000	0.640
0.6	0.10	1.000	0.640
0.7	0.03	0.997	0.640
0.7	0.05	0.997	0.640
0.7	0.10	0.997	0.640

Supplementary Table S2: Per-sample γδ T-cell counts.

Number of gated γδ T-cells (is_gdT = TRUE) per sample. Samples with ≥ 5 gated γδ T-cells were retained for per-sample γδ T-cell summary statistics (Figures 3a and 5c). Whole-atlas and non-γδ T-cell analyses (epithelial HLA expression, atlas-wide chemokine ligand expression) used all 33 tumour samples and all 6 adjacent samples. Samples are sorted by tissue type, then by descending γδ T-cell count.

Sample ID	Tissue type	Gated γδ T-cells	Meets ≥ 5 threshold
GSM6567171_ADJ6	adjacent	121	Yes
GSM6567166_ADJ2	adjacent	56	Yes
GSM6567165_ADJ1	adjacent	45	Yes
GSM6567170_ADJ5	adjacent	18	Yes
GSM6567169_ADJ4	adjacent	11	Yes
GSM6567167_ADJ3	adjacent	2	No
GSM6567157_PDAC1	tumour	126	Yes
GSM6603326_Primary003	tumour	99	Yes
GSM6567160_PDAC3	tumour	84	Yes
GSM8580075_14_P-45_TIL	tumour	75	Yes
GSM6567164_PDAC6	tumour	72	Yes
GSM6567161_PDAC4	tumour	65	Yes
GSM6727544_LPDAC_26	tumour	59	Yes
GSM6567159_PDAC2	tumour	58	Yes
GSM6727548_PDAC_50	tumour	51	Yes
GSM6727543_LPDAC_25	tumour	49	Yes
GSM6727551_PDAC_60	tumour	48	Yes
GSM6567163_PDAC5	tumour	38	Yes
GSM8580076_11_P-44_TIL	tumour	29	Yes
GSM8580078_13_P-42_TIL	tumour	28	Yes
GSM6727550_PDAC_55	tumour	27	Yes
GSM8580088_15_P-46_TIL	tumour	25	Yes
GSM6727547_PDAC_48	tumour	17	Yes
GSM6603324_Primary001	tumour	12	Yes
GSM8580089_16_P-40_TIL	tumour	12	Yes
GSM6727549_PDAC_51	tumour	8	Yes

Sample ID	Tissue type	Gated γδ T-cells	Meets ≥ 5 threshold
GSM6727546_PDAC_47	tumour	7	Yes
GSM8580077_12_P-2_TIL	tumour	7	Yes
GSM6603325_Primary002	tumour	5	Yes
GSM8580083_10_P-32_TIL	tumour	5	Yes
GSM8580080_4_P-20_TIL	tumour	1	No
GSM8580086_7_P-25_TIL	tumour	1	No
GSM8580087_8_P-23_TIL	tumour	1	No
GSM6727545_LPDAC_30	tumour	0	No
GSM8580079_3_P-6_TIL	tumour	0	No
GSM8580081_5_P-33_TIL	tumour	0	No
GSM8580082_1_P-24_TIL	tumour	0	No
GSM8580084_2_P-17_TIL	tumour	0	No
GSM8580085_6_P-3_TIL	tumour	0	No

Supplementary Table S3: Family-wise Benjamini-Hochberg corrected Wilcoxon p-values.

Tumour-versus-adjacent comparisons were performed on per-sample summary statistics using two-sided Wilcoxon rank-sum tests on mean per-sample expression. Raw p-values are reported alongside Benjamini–Hochberg adjusted q-values within each pre-specified gene family. Three HLA class I genes (HLA-B, HLA-A, HLA-E) remained statistically significant after correction (highlighted; $q < 0.05$). KLRC1 in γδ T-cells did not differ significantly between tumour and adjacent tissue at the per-sample level; no checkpoint genes survived family-wise correction.

HLA class I family (per-sample mean in tumour epithelium)

Gene	tumour (n)	adjacent (n)	Median tumour	Median adjacent	Raw p	BH q (family)
HLA-E	33	5	1.06	0.570	0.012	**0.035**
HLA-A	33	5	1.98	1.24	0.018	**0.035**
HLA-B	33	5	2.19	1.34	0.001	**0.007**
HLA-C	33	5	1.74	1.36	0.084	0.126
HLA-F	33	5	0.265	0.175	0.262	0.262
HLA-G	33	5	0.009	0.003	0.192	0.231

Checkpoint family (per-sample mean in γδ T-cells, samples with ≥ 5 cells)

Gene	n tumour	n adjacent	Median tumour	Median adjacent	Raw p	BH q (family)
KLRC1	24	5	0.319	0.414	0.078	0.361
PDCD1	24	5	0.090	0.118	0.750	0.900
TIGIT	24	5	0.199	0.309	0.436	0.817
LAG3	24	5	0.196	0.340	0.544	0.817
HAVCR2	24	5	0.036	0.000	0.120	0.361
CD274	24	5	0.000	0.000	0.908	0.908

Bolded q-values with yellow highlight indicate statistical significance after Benjamini-Hochberg correction within the gene family ($q < 0.05$).

Supplementary Table S4: Master family-wise BH-corrected statistics across the integrated gene family (extended analysis).

Nineteen tumour-versus-adjacent Wilcoxon tests assembled into a single statistical family across four analysis sections: antigen-presentation machinery in epithelium (8 genes), γδ T-cell checkpoint/receptor markers (6 genes), NK cell KLRC1 (1 test), and γδ T-cell chemokine receptors within GSE212966 (4 genes). Each gene/cell-type combination was tested at both the cell level (Wilcoxon on individual cells) and the per-sample level (Wilcoxon on per-sample mean expression; samples with ≥ 10 cells; ADJ5 excluded). Benjamini-Hochberg correction was applied across the unified 19-test family, separately for cell-level and per-sample p-values. log2FC computed as log2((mean_tumour + 0.01) / (mean_adjacent + 0.01)).

Antigen presentation in epithelium (n cells: 52,668 tumour vs 2,931 adjacent; 33 vs 4 samples)

Gene	Mean adj	Mean tum	log2FC	Cell q	Per-sample q
B2M	2.225	3.742	+0.747	< 0.001	0.042
TAP1	0.066	0.213	+1.543	< 0.001	0.042
TAP2	0.043	0.131	+1.411	< 0.001	0.042
HLA-A	1.011	2.178	+1.100	< 0.001	0.071
HLA-B	1.181	2.443	+1.043	< 0.001	0.042
HLA-C	1.158	1.854	+0.674	< 0.001	0.127
HLA-E	0.486	1.067	+1.119	< 0.001	0.067
KLRD1	0.019	0.037	+0.705	0.238	0.240

γδ T-cell checkpoint / receptor markers (n cells: 1,009 vs 235; 19 vs 4 samples)

Gene	Mean adj	Mean tum	log2FC	Cell q	Per-sample q
PDCD1	0.104	0.144	+0.441	0.473	0.968
CD274	0.006	0.008	+0.227	0.645	0.968
KLRC1	0.445	0.268	−0.711	0.003	0.071
HAVCR2	0.029	0.060	+0.856	0.351	0.242
LAG3	0.245	0.220	−0.147	0.159	0.524
TIGIT	0.270	0.246	−0.126	0.238	0.868

NK cell KLRC1 (n cells: 4,056 vs 516; 33 vs 3 samples)

Gene	Mean adj	Mean tum	log2FC	Cell q	Per-sample q
KLRC1	1.424	0.292	−2.246	< 0.001	0.071

γδ T-cell chemokine receptors within GSE212966 (n cells: 443 vs 235; 6 vs 4 samples)

Gene	Mean adj	Mean tum	log2FC	Cell q	Per-sample q
CXCR4	3.047	3.305	+0.117	< 0.001	0.071
CCR6	0.124	0.248	+0.945	0.027	0.190
CCR7	0.338	0.468	+0.457	0.159	0.240
CXCR6	0.448	0.292	−0.602	0.005	0.305

Note: This extended analysis uses ≥ 10 cells per sample with ADJ5 excluded; it complements Supplementary Table S3 (≥ 5 cells, ADJ5 included), the primary statistical framework reported in the manuscript.

Supplementary Table S5: Per-sample malignant epithelial cell classification by inferCNV.

Copy-number inference (inferCNV) was performed sample-wise on the epithelial cluster, with immune and stromal cells from the same sample as the non-malignant reference. n_epi = total epithelial cells per sample considered (capped at 800 where applicable); n_malignant = cells classified as CNV-positive malignant; pct_malignant = malignant percentage; median_cnv = sample median CNV signal. Classification was completed for 38 of 39 samples; GSM6567171_ADJ6 was not classified by inferCNV and is omitted. Adjacent samples retain a minority of CNV-positive cells (median 15.5%, range 6.0–31.2%) while tumour samples are CNV-positive-dominant (median 62.2%, range 10.9–92.9%). Samples sorted by tissue type, then by descending percent malignant.

Sample	Tissue	Dataset	n_epi	n_malignant	% malig	Median CNV
GSM6567170_ADJ5	adjacent	GSE212966	800	250	31.3	0.0017
GSM6567169_ADJ4	adjacent	GSE212966	800	126	15.8	0.0017
GSM6567165_ADJ1	adjacent	GSE212966	650	101	15.5	0.0019
GSM6567167_ADJ3	adjacent	GSE212966	800	55	6.9	0.0012
GSM6567166_ADJ2	adjacent	GSE212966	50	3	6.0	0.0007
GSM6567164_PDAC6	tumour	GSE212966	800	743	92.9	0.0044
GSM8580083_10_P-32_TIL	tumour	GSE279781	60	55	91.7	0.0045
GSM6567159_PDAC2	tumour	GSE212966	800	716	89.5	0.0022
GSM8580079_3_P-6_TIL	tumour	GSE279781	147	126	85.7	0.0034
GSM8580077_12_P-2_TIL	tumour	GSE279781	530	421	79.4	0.0024
GSM6567161_PDAC4	tumour	GSE212966	800	601	75.1	0.0022
GSM8580078_13_P-42_TIL	tumour	GSE279781	800	599	74.9	0.0033
GSM8580076_11_P-44_TIL	tumour	GSE279781	59	44	74.6	0.0039
GSM8580086_7_P-25_TIL	tumour	GSE279781	400	290	72.5	0.0028
GSM6567160_PDAC3	tumour	GSE212966	800	567	70.9	0.0019
GSM6567163_PDAC5	tumour	GSE212966	800	566	70.8	0.0023
GSM6567157_PDAC1	tumour	GSE212966	800	562	70.3	0.0016
GSM6603324_Primary001	tumour	GSE214295	800	562	70.3	0.0032
GSM6603326_Primary003	tumour	GSE214295	800	544	68.0	0.0022
GSM8580084_2_P-17_TIL	tumour	GSE279781	130	86	66.2	0.0032
GSM6727544_LPDAC_26	tumour	GSE217845	668	422	63.2	0.0023

Sample	Tissue	Dataset	n_epi	n_malignant	% malig	Median CNV
GSM6727550_PDAC_55	tumour	GSE217845	800	498	62.3	0.0024
GSM8580075_14_P-45_TIL	tumour	GSE279781	425	240	56.5	0.0024
GSM6727543_LPDAC_25	tumour	GSE217845	522	284	54.4	0.0024
GSM6603325_Primary002	tumour	GSE214295	800	420	52.5	0.0020
GSM6727549_PDAC_51	tumour	GSE217845	800	360	45.0	0.0020
GSM8580087_8_P-23_TIL	tumour	GSE279781	58	26	44.8	0.0020
GSM6727551_PDAC_60	tumour	GSE217845	778	253	32.5	0.0013
GSM8580082_1_P-24_TIL	tumour	GSE279781	495	151	30.5	0.0019
GSM8580089_16_P-40_TIL	tumour	GSE279781	424	129	30.4	0.0018
GSM6727548_PDAC_50	tumour	GSE217845	800	237	29.6	0.0017
GSM6727547_PDAC_48	tumour	GSE217845	800	236	29.5	0.0020
GSM6727545_LPDAC_30	tumour	GSE217845	429	114	26.6	0.0018
GSM6727546_PDAC_47	tumour	GSE217845	800	181	22.6	0.0010
GSM8580081_5_P-33_TIL	tumour	GSE279781	28	5	17.9	0.0016
GSM8580085_6_P-3_TIL	tumour	GSE279781	800	95	11.9	0.0014
GSM8580088_15_P-46_TIL	tumour	GSE279781	800	93	11.6	0.0012
GSM8580080_4_P-20_TIL	tumour	GSE279781	523	57	10.9	0.0013

Supplementary Table S6: HLA-E expression in CNV-confirmed malignant tumour epithelium vs CNV-confirmed non-malignant adjacent epithelium.

Sensitivity analysis restricting the HLA-E per-sample comparison to cells classified by inferCNV: tumour samples contributed only their CNV-positive ("tumour_malig") cells; adjacent samples contributed only their CNV-negative ("adj_normal") cells. This addresses the concern that the headline HLA-E result (Supplementary Table S3) might be confounded by non-malignant epithelium present within tumour samples. Direction is preserved across all comparisons; the cross-cohort per-sample test is borderline (p = 0.062) due to the small adjacent-normal cohort (n = 5). Within the GSE212966 paired cohort, where tumour and adjacent samples come from the same patients, the malignant-restricted comparison reaches p = 0.030.

HLA-E per-sample mean (CNV-restricted)

Comparison	tumour_malig (n)	adj_normal (n)	Mean (T)	Mean (A)	log2FC	Wilcoxon p
All cohorts	33	5	0.937	0.608	+0.63	**0.062**
Within GSE212966 (paired)	6	5	1.115	0.608	+0.96	**0.030**

Per-sample HLA-E expression (full data underlying the test)

Sample	Group	n cells	Mean HLA-E	% positive
GSM6567165_ADJ1	adj_normal	549	0.482	51.7
GSM6567166_ADJ2	adj_normal	47	0.925	70.2
GSM6567167_ADJ3	adj_normal	745	0.294	40.4
GSM6567169_ADJ4	adj_normal	674	0.573	51.2
GSM6567170_ADJ5	adj_normal	550	0.763	60.5
GSM6567157_PDAC1	tumour_malig	562	0.863	58.5
GSM6567159_PDAC2	tumour_malig	716	1.175	62.6
GSM6567160_PDAC3	tumour_malig	567	1.299	68.8
GSM6567161_PDAC4	tumour_malig	601	0.697	51.1
GSM6567163_PDAC5	tumour_malig	566	1.795	82.7
GSM6567164_PDAC6	tumour_malig	743	1.284	84.4
GSM6603324_Primary001	tumour_malig	562	0.944	76.0
GSM6603325_Primary002	tumour_malig	420	1.510	77.4
GSM6603326_Primary003	tumour_malig	544	1.511	79.2
GSM6727543_LPDAC_25	tumour_malig	284	1.219	82.0
GSM6727544_LPDAC_26	tumour_malig	422	0.697	51.9

Sample	Group	n cells	Mean HLA-E	% positive
GSM6727545_LPDAC_30	tumour_malig	114	0.339	23.7
GSM6727546_PDAC_47	tumour_malig	181	0.578	28.7
GSM6727547_PDAC_48	tumour_malig	236	0.688	43.2
GSM6727548_PDAC_50	tumour_malig	237	1.157	80.6
GSM6727549_PDAC_51	tumour_malig	360	0.862	70.8
GSM6727550_PDAC_55	tumour_malig	498	0.765	62.2
GSM6727551_PDAC_60	tumour_malig	253	1.408	61.7
GSM8580075_14_P-45_TIL	tumour_malig	240	1.137	57.9
GSM8580076_11_P-44_TIL	tumour_malig	44	1.253	70.5
GSM8580077_12_P-2_TIL	tumour_malig	421	0.151	12.6
GSM8580078_13_P-42_TIL	tumour_malig	599	0.887	55.1
GSM8580079_3_P-6_TIL	tumour_malig	126	0.579	27.8
GSM8580080_4_P-20_TIL	tumour_malig	57	1.394	47.4
GSM8580081_5_P-33_TIL	tumour_malig	5	1.000	40.0
GSM8580082_1_P-24_TIL	tumour_malig	151	0.546	21.9
GSM8580083_10_P-32_TIL	tumour_malig	55	1.199	70.9
GSM8580084_2_P-17_TIL	tumour_malig	86	0.502	20.9
GSM8580085_6_P-3_TIL	tumour_malig	95	0.638	22.1
GSM8580086_7_P-25_TIL	tumour_malig	290	0.573	23.8
GSM8580087_8_P-23_TIL	tumour_malig	26	0.571	23.1
GSM8580088_15_P-46_TIL	tumour_malig	93	0.909	33.3
GSM8580089_16_P-40_TIL	tumour_malig	129	0.807	50.4

Supplementary Table S7: Per-sample dataset, tissue, and post-QC cell counts.

Sample-level metadata for the integrated PDAC scRNA-seq atlas. All cohorts used the 10X Genomics Chromium single-cell platform. Input was the filtered cell-by-gene count matrices distributed by each GEO submission; uniform QC was applied across all samples (nFeature_RNA ≥ 200, nCount_RNA ≥ 500, percent.mt ≤ 20%). Pre-QC totals are not retained from this pipeline (see Methods M0); they are reported per cohort in the original publications [32, 33, 34, 35]. Adjacent normal samples were available only in GSE212966; the other three cohorts are tumour-only.

Cohort summary

Cohort	Source publication	n samples (T / A)	Post-QC cells
GSE212966	Chen et al., J Transl Med 2023;21:210 [32]	12 (6 / 6)	59,988
GSE214295	Chen et al., Cancer Lett 2024;576:216586 [35]	3 (3 / 0)	22,934
GSE217845	Caronni et al., Nature 2023;623:415–422 [33]	9 (9 / 0)	58,596
GSE279781	Montagne et al., iScience 2025;28:111569 [34]	15 (15 / 0)	108,951
**Total**		**39 (33 / 6)**	**250,469**

Per-sample breakdown (n = 39)

Sample ID	Cohort	Tissue	Post-QC cells
GSM6567171_ADJ6	GSE212966	adjacent	6,256
GSM6567166_ADJ2	GSE212966	adjacent	6,101
GSM6567165_ADJ1	GSE212966	adjacent	4,214
GSM6567169_ADJ4	GSE212966	adjacent	4,033
GSM6567170_ADJ5	GSE212966	adjacent	3,041
GSM6567167_ADJ3	GSE212966	adjacent	1,378
GSM6567157_PDAC1	GSE212966	tumour	7,264
GSM6567161_PDAC4	GSE212966	tumour	6,223
GSM6567159_PDAC2	GSE212966	tumour	5,960
GSM6567160_PDAC3	GSE212966	tumour	5,936
GSM6567164_PDAC6	GSE212966	tumour	4,986
GSM6567163_PDAC5	GSE212966	tumour	4,596
GSM6603325_Primary002	GSE214295	tumour	10,894
GSM6603326_Primary003	GSE214295	tumour	8,649
GSM6603324_Primary001	GSE214295	tumour	3,391

Sample ID	Cohort	Tissue	Post-QC cells
GSM6727546_PDAC_47	GSE217845	tumour	12,914
GSM6727550_PDAC_55	GSE217845	tumour	8,064
GSM6727548_PDAC_50	GSE217845	tumour	7,998
GSM6727549_PDAC_51	GSE217845	tumour	7,733
GSM6727547_PDAC_48	GSE217845	tumour	6,481
GSM6727544_LPDAC_26	GSE217845	tumour	4,922
GSM6727545_LPDAC_30	GSE217845	tumour	4,817
GSM6727551_PDAC_60	GSE217845	tumour	3,145
GSM6727543_LPDAC_25	GSE217845	tumour	2,522
GSM8580075_14_P-45_TIL	GSE279781	tumour	45,188
GSM8580088_15_P-46_TIL	GSE279781	tumour	16,461
GSM8580076_11_P-44_TIL	GSE279781	tumour	12,032
GSM8580078_13_P-42_TIL	GSE279781	tumour	10,411
GSM8580089_16_P-40_TIL	GSE279781	tumour	9,648
GSM8580077_12_P-2_TIL	GSE279781	tumour	3,595
GSM8580086_7_P-25_TIL	GSE279781	tumour	1,766
GSM8580085_6_P-3_TIL	GSE279781	tumour	1,684
GSM8580079_3_P-6_TIL	GSE279781	tumour	1,506
GSM8580083_10_P-32_TIL	GSE279781	tumour	1,316
GSM8580081_5_P-33_TIL	GSE279781	tumour	1,277
GSM8580080_4_P-20_TIL	GSE279781	tumour	1,095
GSM8580087_8_P-23_TIL	GSE279781	tumour	1,037
GSM8580082_1_P-24_TIL	GSE279781	tumour	999
GSM8580084_2_P-17_TIL	GSE279781	tumour	936
